# Genome-wide identification and analysis of *RING finger* gene family and the self-compatibility-associated *SBP1* gene in goji berry (*Lycium barbarum*)

**DOI:** 10.3389/fpls.2026.1863389

**Published:** 2026-06-30

**Authors:** Zhonghua Wang, Jiali Wu, Zijun Yang, Chenglin Zhao, Yajing An, Cuiping Wang

**Affiliations:** 1 School of Biological Science and Engineering, North Minzu University, Yinchuan, Ningxia, China; 2 Ningxia Grape and Wine Innovation Center, North Minzu University, Yinchuan, Ningxia, China

**Keywords:** LbSBP1, *Lycium barbarum*, RING finger gene family, self-compatibility, yeast two-hybrid

## Abstract

RING finger proteins are a large family of E3 ubiquitin ligases that are crucial for modulating plant growth, development, and biotic and abiotic stress responses. The RING-HC-type SBP1 protein serves as a core component of the SCFSLF/SFB ubiquitin ligase complex and modulates gametophytic self-incompatibility (GSI) in plants. As a typical GSI species, *Lycium barbarum* remains poorly characterized regarding its *RING finger* gene family and *SBP1* homologous genes. Here, we systematically identified 213 *RING finger* genes at the genome-wide level in *L. barbarum*. Based on phylogenetic relationships, gene structures, conserved motifs, and domain features, these genes were categorized into seven subfamilies covering RING-H2, RING-HC, and RING-v subtypes. Chromosomal localization analysis revealed that all *RING finger* genes are distributed on 12 chromosomes, including 10 tandem duplication pairs and 50 segmental duplication pairs. An *SBP1* homologous gene that exhibits high sequence similarity with SBP1 proteins from other Solanaceae species was screened from the *L. barbarum RING finger* family. This homolog is highly and specifically expressed in pollen and encodes a nuclear-localized protein. Yeast two-hybrid assays verified its physical interaction with multiple male GSI determinant proteins in *L. barbarum*, namely *S_2_-LbSLF7*, *S_2_-LbSLF9*, *S_2_-LbSLF11*, *S_2_-LbSLF12*, and *S_2_-LbSLF13*. This study comprehensively characterizes the *RING finger* gene family in *L. barbarum* and identifies a GSI-associated *SBP1* homolog that potentially regulates pollen development and self-incompatibility. Our results provide a vital foundation for uncovering the molecular mechanism of wolfberry GSI and offer valuable genetic resources for the molecular breeding of self-compatible *L. barbarum* varieties.

## 1 Introduction

*Lycium barbarum* is a perennial deciduous shrub belonging to the genus *Lycium* within the family Solanaceae. The genus *Lycium* comprises approximately 80 species distributed across the Americas and Asia, while *L. barbarum* is specifically adapted to the arid and semi-arid regions of northwestern China. As a traditional economic crop, it has a long history of both medicinal and dietary applications. *L. barbarum* is the primary source of goji berries, which are rich in a diverse array of nutritional components, including vitamins, polysaccharides (LBP), carotenoids, and betaine. These compounds confer significant physiological benefits on the plant, such as antioxidant, anti-aging, anti-tumor, and immunomodulatory properties, thereby garnering extensive attention and demonstrating broad market prospects (Zhang et al., 2025; Liu et al., 2024, 2025). Ningxia is recognized as the genuine producing region for *L. barbarum*, with a cultivation history spanning over 500 years (Ma et al., 2019). Consequently, the production of this crop has long served as a crucial driving force for the economic development of this region.

Ubiquitin (Ub), a small signaling protein composed of 76 amino acids, was first discovered by Goldstein in bovine thymus in 1975. As a highly stable and conserved molecule ubiquitously distributed across tissues, it is essential for maintaining protein homeostasis in eukaryotes (Peng et al., 2023). The covalent attachment of Ub to substrate proteins via a three-step enzymatic cascade (activation, conjugation, and ligation) is defined as ubiquitination (Ji et al., 2023; de Heiden and Mulder, 2025). As a ubiquitous post-translational modification mechanism, ubiquitination requires the synergistic actions of ubiquitin-activating enzymes (E1), ubiquitin-conjugating enzymes (E2), and Ub ligases (E3). This cascade drives the efficient and specific transfer of Ub to target substrates, thereby exerting extensive regulatory functions in diverse cellular processes (Ciechanover, 2015; Brillada and Trujillo, 2022; Zhang et al., 2024). Really Interesting New Gene(RING) finger proteins constitute a vast and highly diverse family of zinc finger proteins, the majority of which function as E3 Ub ligases. By mediating the covalent binding of ubiquitin to substrates to regulate protein degradation, these proteins play indispensable roles in critical biological processes, including DNA repair and mitochondrial protein quality control (Liu et al., 2022). Based on the nature of the fifth metal-coordinating residue, RING finger domains are classified into either canonical or variant types. The canonical types include RING-H2 (with cysteine as the fifth ligand) and RING-HC (with histidine as the fifth ligand), whereas the variant types encompass RING-C2, RING-v, RING-D, RING-S/T, and RING-G (Stone et al., 2005). Lorick et al. (1999) demonstrated that the RING-H2 and RING-HC domains are crucial for catalyzing the E3 ligase activity of RING-containing proteins. In plants, RING finger proteins have been implicated in various biological functions. For instance, a putative RING-HC protein, *ZmRHCP1*, was identified in maize, which functions in lateral root initiation and exhibits expression regulated by abiotic stresses (Li et al., 2017). In *Arabidopsis thaliana*, the *RING finger* family member *SINAT5* activates the ubiquitin-proteasome system to target the transcription factor *NAC1* for ubiquitination and subsequent degradation, thereby negatively regulating the growth and development of primary and lateral roots (Fang et al., 2023). Similarly, the maize RING-H2 proteins ZmXerico1/2 participate in the regulation of drought stress responses by stabilizing ABA 8’-hydroxylase to attenuate ABA degradation (Brugière et al., 2017). Furthermore, the *A. thaliana* RING-H2 gene *BRH1* is involved in the brassinosteroid signaling transduction pathway to modulate developmental processes such as leaf expansion and cell proliferation, ultimately positively regulating leaf morphogenesis and leaf size (Wang et al., 2018).

Self-incompatibility (SI) is a highly conserved reproductive barrier mechanism in flowering plants, wherein pistil tissues (e.g., the stigma or style) specifically recognize self-pollen and consequently inhibit pollen tube germination or growth. This effectively prevents self-fertilization and promotes outcrossing, thereby ensuring the genetic diversity of populations. This SI response is governed by genetic polymorphism at the S-locus and is broadly classified into gametophytic (GSI) and sporophytic (SSI) systems (Vekemans and Castric, 2021; Hu et al., 2025). In GSI, the rejection response is dictated by the haploid genotype of the pollen grain. Although self-pollen typically germinates on the stigma and penetrates the style, its tubes ultimately swell, curl, and arrest their growth within the style (Nettancourt, 1997). In solanaceous plants, GSI operates via an S-RNase-based mechanism. The stylar S-determinant is encoded by a single polymorphic glycoprotein with ribonuclease activity (S-RNase), whereas the pollen S-determinant is collectively encoded by multiple polymorphic genes containing an F-box domain (S-locus F-box, SLFs) (Lee et al., 1994; Kubo et al., 2010). These SLF proteins assemble with Cullin1, SKP1, and Rbx1 to form an SCF complex, which specifically recognizes and binds non-self S-RNases, targeting them for ubiquitination and subsequent degradation (Entani et al., 2014). *L. barbarum* is a typical GSI species, with the majority of contemporary cultivars exhibiting SI (Wu et al., 2025). The SI trait exerts severe detrimental impacts on large-scale monoculture. Reproductive disorders induced by SI can lead to excessive flowering, physiological fruit abscission, severe morphological differentiation of fruits, and a drastic reduction in the fruit-setting rate, which collectively compromise the yield and quality of goji berry production (Wang et al., 2024). Elucidating the underlying mechanisms of SI in *L. barbarum* is therefore of critical importance for its molecular breeding, genetic improvement, and the development of highly efficient production systems.

SBP1 (S-RNase binding protein 1) encodes a protein containing a RING-HC domain and serves as a core component of the SCF^SLF/SFB^ E3 ubiquitin ligase complex. Representing a class of plant-specific RING finger E3 ubiquitin ligases, SBP1 non-specifically binds to S-RNases and regulates self-pollen recognition and degradation via the ubiquitination pathway, thereby actively participating in SI regulation. Sims and Ordanic (2001) first identified the *SBP1* gene in *Petunia hybrida*, demonstrating that its encoded protein, which harbors a canonical RING-HC domain, is involved in the SI pathway. This finding provided crucial molecular evidence for elucidating the functional mechanisms of SBP1 in GSI. Furthermore, Martin et al. demonstrated that SBP1 likely acts as the RING domain functional module within the E3 ubiquitin ligase complex. Through its conserved RING-H2 zinc finger conformation, SBP1 specifically recognizes S-RNases and mediates their ubiquitination and subsequent degradation; moreover, this RING finger protein may confer specificity on the ubiquitination complex (O’Brien et al., 2004).

In recent years, significant progress has been made in understanding E3 ubiquitin ligases, SI mechanisms, and SBP1 functions within the Solanaceae family. SBP1 itself is a RING-HC domain-containing E3 ubiquitin ligase that acts as a core component of the SCF^SLF^ complex in GSI, mediating the ubiquitin-mediated degradation of non-self S-RNase. The functional repertoire of SBP1 has expanded from reproductive regulation to immune modulation: in tomato, a prominent member of the Solanaceae family, SBP1 acts as an E3 ligase to dynamically maintain the proteostasis of the NLR immune receptor Sw-5b. In the absence of pathogen effectors, SBP1 mediates the gradual turnover of autoinhibited Sw-5b; however, upon invasion by tomato spotted wilt virus effector protein NSm, NSm competitively disrupts the SBP1-Sw-5b interaction, thereby stabilizing the activated Sw-5b and triggering a robust immune response (Wang et al., 2024). Regarding the SI mechanism, in addition to the aforementioned SCF^SLF^-mediated ubiquitination-degradation pathway, studies by Tian et al. and Xue have uncovered a parallel mechanism: during self-pollination, S-RNase undergoes liquid-liquid phase separation (LLPS) via its intrinsically disordered regions (IDRs) to form S-RNase condensates (SRCs). Facilitated by HT-B and thioredoxin h, these SRCs sequester the actin-binding protein PhABRACL, disrupting the cytoskeleton and inhibiting pollen tube growth. This integrates “ubiquitin-mediated degradation” and “phase separation-driven condensation” into a dual molecular model of the SI system (Tian et al., 2024; Xue, 2025). Furthermore, breakthroughs have been achieved regarding other types of E3 ubiquitin ligases in Solanaceae disease resistance: in eggplant, the CUL4-DDB1-DDA1 E3 ligase complex positively regulates bacterial wilt resistance by specifically degrading the transcription factor SmNAC via the substrate receptor SmDDA1b. Additionally, the SmDDB1 protein interacts with both SmCUL4 and SmDDA1b, thereby expanding our understanding of the regulatory mechanisms of plant E3 ligases (Yan et al., 2024). These molecular models provide a crucial theoretical framework for understanding the function of LbSBP1 in *L. barbarum* SI.

In this study, we conducted a genome-wide identification of the *RING finger* gene family in *L. barbarum*. To establish a comprehensive profile of this family, the chromosomal distributions, gene structures, protein physicochemical properties, conserved motifs, and domains of its members were systematically characterized. The *SBP1* gene was subsequently retrieved from this family based on phylogenetic and sequence alignment analyses. Furthermore, tissue-specific expression patterns and subcellular localization of LbSBP1 were evaluated, and its interaction with SLF—the pollen S-determinant governing SI in *L. barbarum*—was extensively investigated. Collectively, these analyses lay a solid theoretical foundation for further elucidating the potential role of LbSBP1 in the SI recognition mechanism.

## 2 Materials and methods

### 2.1 Sample harvesting and processing

*L. barbarum* ‘Ningqi 1’ used in this study was cultivated at the goji germplasm nursery of the Yinchuan Botanical Garden, Ningxia, China (38°25′N, 105°10′E). During the peak flowering season in June 2025, three 10-year-old plants of uniform growth vigor were selected, with each individual plant serving as a biological replicate. Samples of young leaves, petals, whole flowers, styles, pedicels, and pollen collected from the current-year branches of each plant were immediately frozen in liquid nitrogen, and stored at -80°C for subsequent analyses.

### 2.2 Identification of *RING finger* gene family members in *Lycium barbarum*

The whole-genome sequence of *L. barbarum* was retrieved from NCBI (assembly ASM1917538v2, BioProject PRJNA640228). Using known *A. thaliana* RING finger proteins as queries, the coding sequences (CDSs) of *L. barbarum RING finger* genes were extracted from the genome annotation file (GFF3 format) using TBtools (Chen et al., 2020), and protein sequences were obtained via the built-in translation function. To ensure sequence reliability, sequence identifiers were simplified, and a reciprocal BLAST analysis was performed to eliminate redundant sequences. The conserved domains of the obtained protein sequences were analyzed using the SMART (Letunic et al., 2021) and NCBI CDD (Yang et al., 2020) databases. Furthermore, the physicochemical properties of RING finger proteins were evaluated using the ExPASy (Wilkins et al., 1999), and subcellular localizations were predicted via Plant-mPLOC (http://www.csbio.sjtu.edu.cn/bioinf/plant-multi/).

### 2.3 Promoter analysis and Cis-acting element prediction

To investigate the *cis*-acting elements within the promoter regions of the *L. barbarum RING finger* genes, the 2000-bp sequences upstream of the start codon (ATG) for all 213 identified genes were extracted using TBtools. These putative promoter sequences were submitted to the PlantCARE database (https://bioinformatics.psb.ugent.be/webtools/plantcare/html/) for the identification of *cis*-acting elements. The annotation results were then visualized and categorized using the “PlantCARE Result Classify” function in TBtools (Bailey et al., 2009). The *cis*-elements associated with light responsiveness, phytohormone regulation, stress responsiveness, and growth and development were selected for comparative analyses.

### 2.4 Analysis of conserved motifs, domains, and gene structures

To identify the conserved motifs within the 213 RING finger proteins, the MEME online tool (https://meme-suite.org/meme/index.html) was employed, with the maximum number of motifs set to 20 and other parameters kept at default settings (Bailey et al., 2009). The conserved domains of protein sequences were characterized using the NCBI CDD (Yang et al., 2020) and SMART (Letunic et al., 2021) databases. Based on the genome annotation of *L. barbarum*, the intron-exon organizations of the *RING finger* were mapped using TBtools. TBtools was utilized to generate a schematic diagram illustrating the motif distributions, conserved domains, gene structures, and the phylogenetic tree.

### 2.5 Chromosomal localization, collinearity, and evolutionary selection pressure analysis

The chromosomal location of the 213 *RING finger* genes was extracted from the *L. barbarum* genome annotation, and their distribution map was generated using TBtools (Chen et al., 2006). To identify gene duplication events within this family, including segmental and tandem duplications, the MCScanX module in TBtools was employed. An inter-species syntenic analysis was performed to compare the *RING finger* genes of *L. barbarum* with those of *A. thaliana*, *S. lycopersicum*, and *N. tabacum*. The results were visualized using the Advanced Circos tool in TBtools (Bailey et al., 2009). To evaluate the evolutionary selection pressures, the synonymous (*K*s), non-synonymous (*K*a) substitution rates, and their *K*a/*K*s ratios were calculated for intra-species tandemly and segmentally duplicated genes, as well as for inter-species orthologous gene pairs (Gao et al., 2018).

### 2.6 Phylogenetic analysis of the *RING finger* family in *L. barbarum*

To elucidate the evolutionary relationships of the 213 *L. barbarum RING finger* members identified in this study, multiple sequence alignment of their protein sequences was performed using ClustalW implemented in MEGA 11 (Tamura et al., 2021). A phylogenetic tree was subsequently constructed based on the Neighbor-Joining (NJ) method with the Poisson correction model. The node reliability was assessed with 1000 bootstrap replicates. Finally, the generated phylogenetic tree was visualized and graphically refined using the Interactive Tree Of Life online platform (https://itol.embl.de/upload.cgi) (Letunic and Bork, 2019).

### 2.7 Identification of SBP1 in *L. barbarum*

To investigate whether the *L. barbarum* genome harbors the *SBP1* gene, SBP1 protein sequences from *Malus domestica* (*MdSBP1*: BAO04295.1), *Prunus avium* (*PaSBP1*: AGW01013.1), *N. alata* (*NaSBP1*: ACD40009.1), *Populus alba* (*PoaSBP1*: TKR78833.1), *P. hybrida* (*PhSBP1*: AAF28357.2), and *Petunia inflata* (*PiSBP1*: ABB77434.1) were retrieved from the NCBI database as reference sequences. Given that the lengths of these reference SBP1 proteins range from 330–350 amino acids, the screening threshold was broadened to 290–380 amino acids to account for natural variations. Candidate proteins falling within this specified length range were extracted from the 213 *L. barbarum RING finger* members. An NJ phylogenetic tree was constructed utilizing these candidates alongside the six reference sequences, employing the Poisson correction model, with branch support evaluated using 1000 bootstrap replicates. The *L. barbarum* RING finger proteins clustering with the reference SBP1s were selected for homology validation via the NCBI BLAST tool (https://blast.ncbi.nlm.nih.gov/Blast.cgi?PROGRAM=blastp&PAGE_TYPE=BlastSearch&LINK_LOC=blasthome).

### 2.8 Expression analysis of *RING finger* genes

Based on the phylogenetic clustering results, nine *L. barbarum RING finger* genes exhibiting the closest evolutionary relationship with the SBP1 proteins were selected as candidates. Gene-specific primers (**Table 1**) were designed using Primer Premier 5.0 based on coding sequences (CDSs) and synthesized by Sangon Biotech (Shanghai, China). Total RNA was extracted from the young leaves, petals, whole flowers, styles, pedicels, and pollen of the ‘Ningqi 1’ cultivar, and reverse-transcribed into cDNA for quantitative reverse transcription PCR (qRT-PCR) analysis. The *L. barbarum Actin* gene was employed as the reference. The 10 μL qRT-PCR mixture comprised 5 μL of 2×TransTaq^®^ HiFi PCR Super Mix II, 0.4 μL each of forward and reverse primers, 0.5 μL of cDNA template, and 3.7 μL of ddH_2_O. The thermal cycling conditions were as follows: an initial denaturation step at 95°C for 30 s, followed by 40 cycles of denaturation at 95°C for 5 s and annealing/extension at 60°C for 30 s. All reactions were performed with three biological replicates. The relative expression levels of the target genes were calculated using the 2^−ΔΔCt^ method (Livak and Schmittgen, 2001). Statistical significance of the expression differences among tissues was determined by one-way analysis of variance (ANOVA) using GraphPad Prism 9.5, and visualized as bar graphs.

**Table 1:** Primers used for quantitative real-time PCR.

Gene Name	Forward primer(5’∼3’)	Reverse primer(5’∼3’)
rna-XM_060357446.1	TCTTCTCAATCATCAGCCTTCT	TATTGTTTCCTCGGCGGTTA
rna-XM_060343438.1	TGATGGAGGGCTGGATTTGC	CGACCGAGGCTGCAATAGAT
rna-XM_060348651.1	TTTGAACAGCAAGAACAGGGAA	CAGGGCATTGACAACCGACT
rna-XM_060351514.1	GCCTCAACCGCAACTCATTG	CGGTGGAGACAACATTCGGA
rna-XM_060357984.1	ACGAGGCAGGGAAGTAGAAG	TTGCGGAACTGACAGAAGAG
rna-XM_060319712.1	CAGGTCGCATCAAGGAAACG	TACTGTTGAACCAGCGGCAA
rna-XM_060319711.1	TGTTAGCATGGGGAGATGCG	CACAAATGCCTGCACGGTAA
rna-XM_060323896.1	ATCCAGGAGTTGAAGCGAATT	CGATGAGTTGCGGTTGAGAT
rna-XM_060337022.1	GAGTCTTGTTGTGGCAGCAG	CAACACGCACGATTCCCTCT
LbActin	CTCAGCACCTTCCAGCAGAT	TAACACTGCAACCGCATTTC

### 2.9 Subcellular localization of LbSBP1

To determine the subcellular localization of LbSBP1, the cloned *LbSBP1* was inserted into the pBWA(V)H2STMVΩ-eGFP vector via homologous recombination to generate the pBWA(V)H2STMVΩ-LbSBP1-eGFP fusion expression vector. The validated recombinant plasmid was introduced into *Agrobacterium tumefaciens* GV3101 via electroporation, followed by incubation at 30 °C for 2 days. A single colony was then inoculated into liquid Yeast Extract Beef medium supplemented with the appropriate antibiotics and grown at 28°C with shaking at 170 rpm until the logarithmic growth phase. Bacterial cells were harvested by centrifugation and resuspended in an infiltration buffer (10 mM MgCl_2_, 120 μM acetosyringone) to an OD_600_ of approximately 0.6. The bacterial suspension was infiltrated into the abaxial epidermis of healthy, one-month-old *N. benthamiana* plants using a needleless syringe (Wang et al., 2021). The plants were maintained in low-light conditions for 48 h. Transient sections of the infiltrated young leaves were then prepared, and the eGFP fluorescence signals were observed using a Nikon C2-ER laser scanning confocal microscope (Nikon, Tokyo, Japan) with an excitation wavelength of 488 nm and an emission wavelength of 510 nm (Liu et al., 2018). To further validate the localization specificity, organelle marker plasmids were transformed into *Agrobacterium*. The bacterial suspensions were mixed with the *LbSBP1-eGFP* suspension at a 1:1 ratio and co-infiltrated into *N. benthamiana* leaves. The subsequent procedures were identical, and dual-channel fluorescence signals were captured and overlaid using the confocal microscope.

### 2.10 Yeast two-hybrid analysis of SBP1–SLF interactions

To investigate the protein-protein interactions between LbSBP1 and the S2-LbSLF proteins, a yeast two-hybrid (Y2H) assay was performed (Feng et al., 2021). The full-length coding sequences of *LbSBP1* and the 13 *S2-LbSLFs* were cloned into the pGADT7 (AD) and pGBKT7 (BD) vectors, respectively. The AD-*LbSBP1* construct was co-transformed with each of the 13 BD-*S2-LbSLF* constructs into the yeast strain Y2HGold. The pGBKT7-53/pGADT7-T and pGBKT7-Lam/pGADT7-T combinations served as positive and negative controls, respectively. The co-transformants were initially selected on synthetic dropout (DDO; SD/-Leu/-Trp) medium containing 2% agar. Well-growing colonies were then spotted onto DDO, quadruple-dropout (QDO; SD/-Leu/-Trp/-His/-Ade), and QDO/X-α-Gal/AbA plates to evaluate any auto-activation of the foreign proteins and to assess the interactions. Yeast cultures were subjected to 10-fold serial dilutions with sterile water to generate three consecutive dilutions, which were then spotted onto the plates. Following incubation at 30°C for 3–4 days, colony growth was photographically documented.

## 3 Results

### 3.1 Identification and physicochemical characterization of the *RING finger* gene family in *L. barbarum*

In total, we identified 213 *RING finger* genes from the *L. barbarum* genome, and their physicochemical properties were systematically characterized (**Supplementary Table 1**). The amino acid lengths of these proteins varied significantly, ranging from 149–884 residues, with corresponding molecular weights (MWs) spanning 16.85–101.00 kDa. Based on sequence length, the members were distributed across three intervals: the majority comprised 301–600 amino acids (105 members), followed by 0–300 amino acids (92 members), and 601–1000 amino acids (16 members). The predicted theoretical isoelectric points ranged from 3.98–10.01, with most proteins (130) being acidic (pI < 7) and the remaining 83 being basic (pI > 7). Furthermore, hydrophilicity analysis (Grand Average of Hydropathy) indicated that the vast majority of members (183) were hydrophilic, whereas only 30 exhibited hydrophobic characteristics. Subcellular localization prediction revealed a strong nuclear preference, with 202 members (94.3%) predicted to localize to the nucleus; the remainder were distributed in either the mitochondria (10) or the cell membrane (1). Based on their conserved domains, these RING finger proteins were classified into three subtypes: RING-H2 (133, 62.44%), RING-HC (64), and RING-v (16), with the latter two collectively accounting for 37.56% of the family. Overall, these findings indicate that, despite considerable variability in basic physicochemical properties, the *L. barbarum RING finger* gene family exhibits distinct preferences regarding subcellular localization and domain architecture.

### 3.2 *Cis*-acting element analysis of the *RING finger* gene family in *L. barbarum*

**Figure 1:**
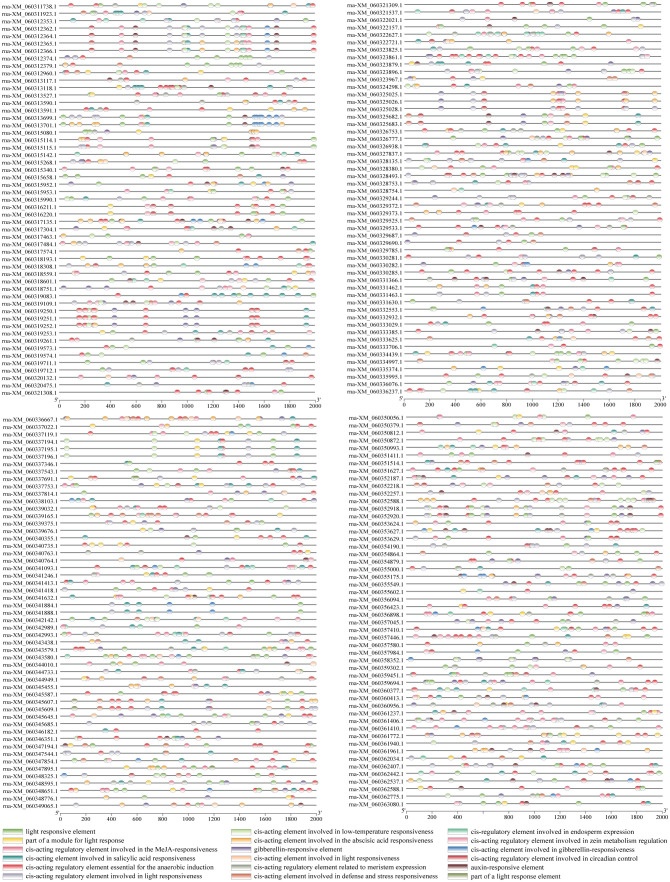
Analysis of cis-acting elements in the *RING finger* gene family of *L. barbarum*. The 2000-bp sequences upstream of the translation start codon of the *RING finger* genes were extracted for the analysis. Different colored boxes represent distinct types of *cis*-acting elements, while black lines indicate the length of the promoter regions.

To elucidate the regulatory potential of the *L. barbarum RING finger* genes, the *cis*-acting elements within their promoter regions were investigated (**Figure 1**). A total of 4,618 *cis*-acting elements, classified into 18 distinct types, were identified across these promoter sequences. These elements were classified into four major functional categories: light responsiveness, phytohormone regulation, stress responsiveness, and growth and development. Among these, light-responsive elements were the most abundant, comprising 2,320 occurrences (50.24% of the total), encompassing various light-responsive motifs and modules. Phytohormone-responsive elements constituted the second largest category (1,338; 28.97%), including *cis*-acting elements associated with methyl jasmonate (MeJA), abscisic acid (ABA), salicylic acid (SA), gibberellin (GA) and auxin responsiveness. Stress-responsive elements accounted for 678 instances (14.68%), primarily involving motifs involved in low-temperature, anaerobic induction, and general defense and stress responses. Finally, elements associated with growth and development represented the smallest fraction (282; 6.11%), containing motifs related to zein metabolism regulation, circadian control, endosperm expression, and meristem expression. Collectively, these findings demonstrate that the promoters of the *L. barbarum RING finger* genes are highly enriched with regulatory elements associated with light, phytohormone, and stress responses, strongly suggesting that this gene family may play pivotal roles in diverse physiological processes, including photosynthetic regulation, hormone signal transduction, and abiotic stress responses in *L. barbarum.*

### 3.3 Integrated analysis of motifs, domains, and gene structures of the *RING finger* gene family in *L. barbarum*

**Figure 2:**
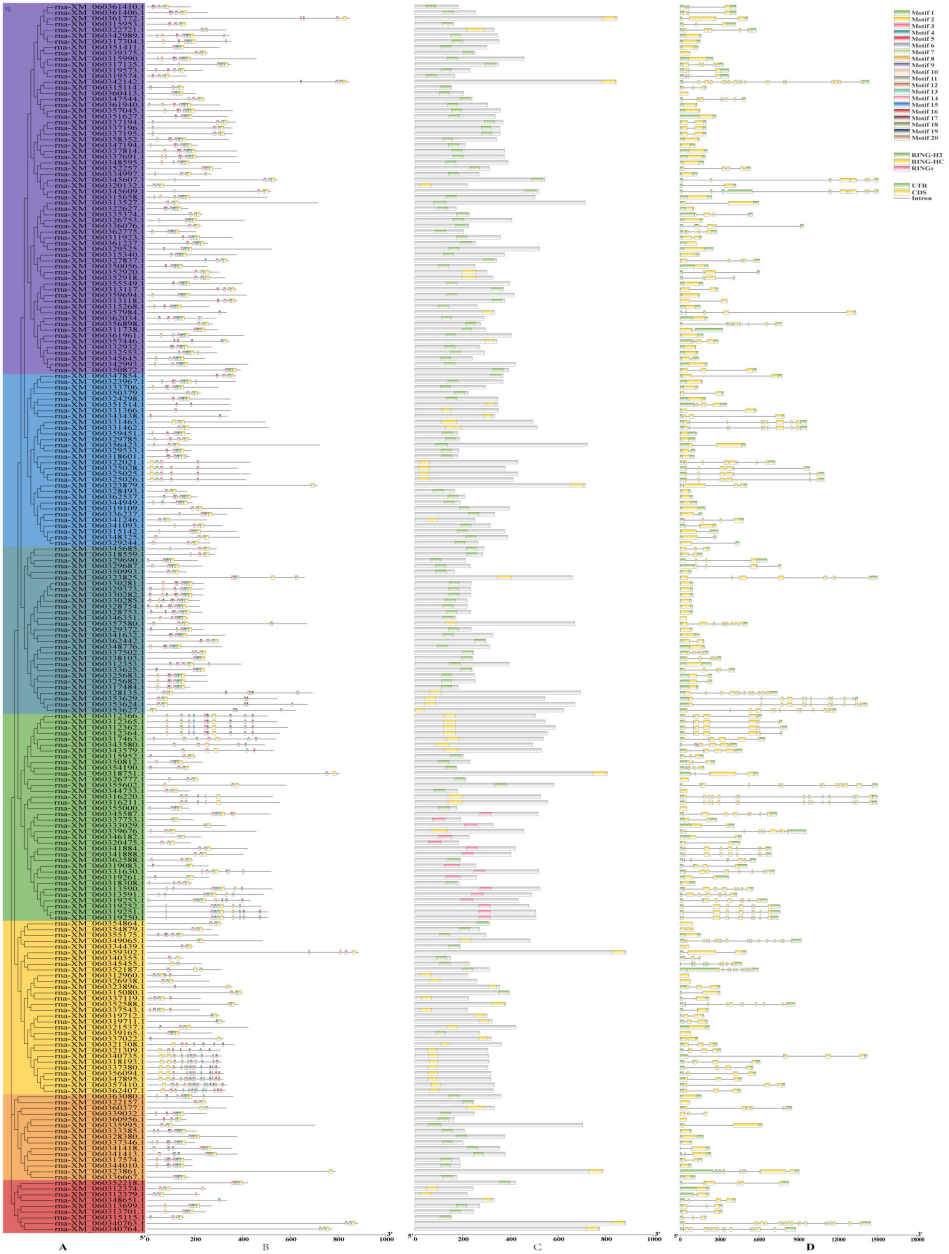
Phylogenetic tree, motifs, domains, and gene structure of RING finger proteins in *L. barbarum*. **(A)** Phylogenetic tree of 213 RING finger proteins constructed using the Neighbor-Joining method. Different subgroups are distinguished by different background colors and labels. **(B)** Conserved motifs of RING finger proteins. Different motifs are represented by colored boxes. **(C)** Conserved domains of RING finger proteins. Different domains are highlighted in different colors. **(D)** Gene structures of *RING finger* genes. Exons, introns, and UTRs are represented by yellow boxes, black lines, and green boxes, respectively.

A comprehensive analysis of the phylogenetic relationships, conserved motifs, conserved domains, and gene structures of the *L. barbarum RING finger* gene family was performed using TBtools (**Figure 2**). The conserved motif analysis revealed that each of the 213 proteins contained 1–15 motifs. Notably, motif 2 was present in 207 proteins, highlighting its high conservation within the family; motifs 1, 3, and 4 were also relatively well-conserved across the majority of the members. An integration of the phylogenetic tree with motif distribution patterns demonstrated that Group III harbored the highest diversity of motif types (19), whereas Group I contained the fewest (7). Groups II, IV, V, VI, and VII possessed 13, 16, 15, 16, and 17 distinct motif types, respectively. Proteins within the same clade generally shared highly similar or identical motif compositions and arrangements, implying functional conservation and close evolutionary relationships. Furthermore, an examination of the conserved domains revealed an uneven distribution of the RING-H2, RING-HC, and RING-v subtypes across the seven phylogenetic groups. Specifically, the number of RING-H2 members in Groups I through VII was 4, 13, 9, 9, 23, 19, and 56, respectively, while the RING-HC members were distributed as 5, 2, 21, 11, 6, 11, and 8. Notably, the RING-v subtype was exclusively restricted to Group IV, comprising 16 members. Although no strict one-to-one correspondence was observed between the 20 identified motifs and the three domain types, members within individual clades still exhibited a high degree of consistency in both their motif repertoires and domain architectures. Finally, analysis of the gene structures revealed that the 213 genes possessed 1 to 19 exons and 0 to 18 introns. The vast majority of these genes contained untranslated regions (UTRs), with only 15 genes lacking UTR sequences.

### 3.4 Chromosomal distribution, synteny, and evolutionary selection analysis of the *RING finger* gene family in *L. barbarum*

**Figure 3:**
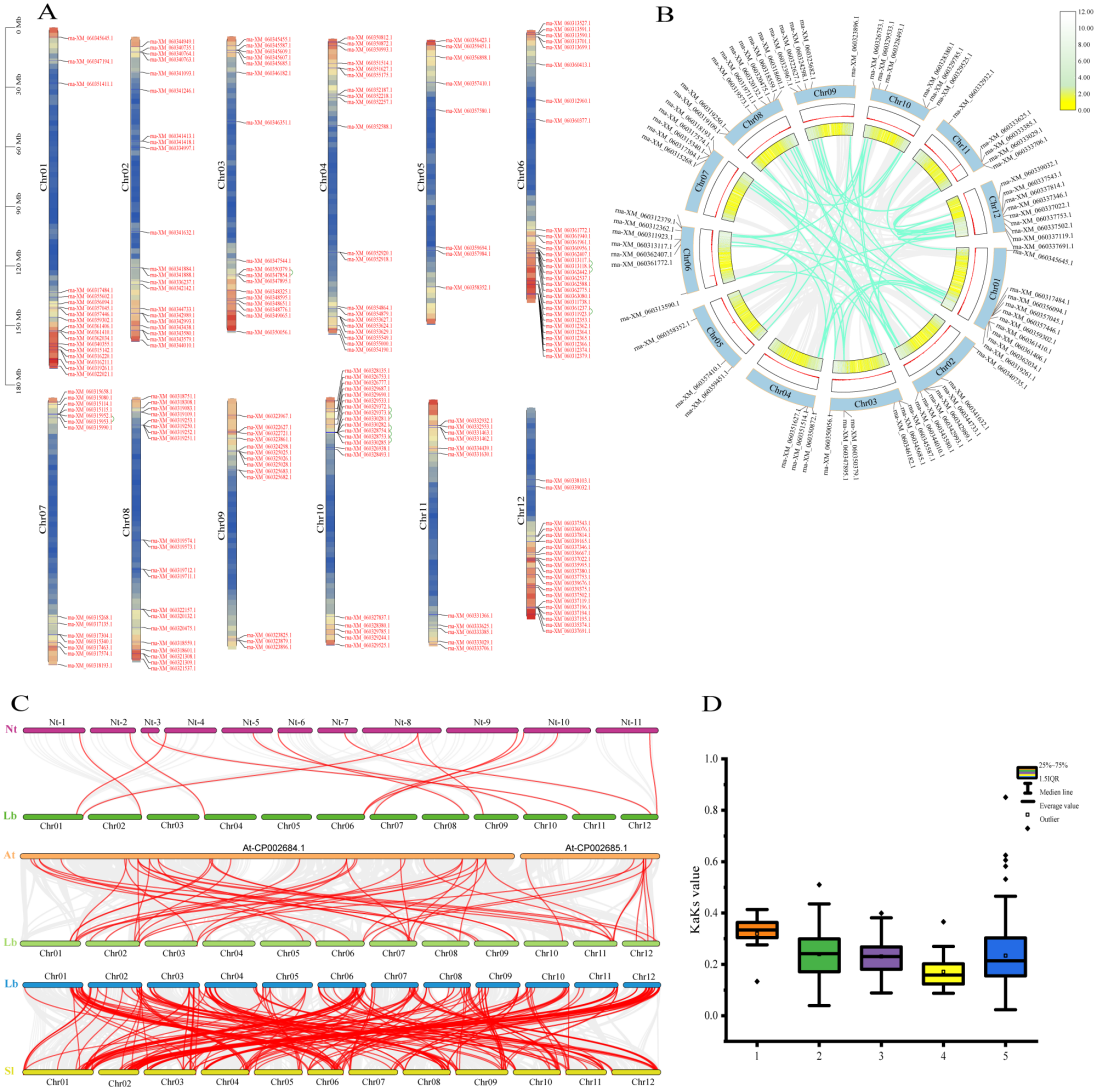
Chromosomal distribution, synteny, and selection pressure analysis of RING finger proteins in *L. barbarum*. **(A)** Chromosomal locations of the 213 *RING finger* genes. “Chr” denotes chromosome, and the vertical scale represents chromosome length (Mb). Green connecting lines indicate tandem duplicate gene pairs. **(B)** Intraspecific synteny analysis of *RING finger* genes within the *L. barbarum* genome. The positions and names of segmentally duplicated genes are marked on the chromosomes. Green lines in the center represent syntenic *RING finger* gene pairs, while gray lines indicate collinear blocks within the genome. “Chr” denotes chromosome. **(C)** Interspecific synteny analysis of *RING finger* genes between *L. barbarum* and three other species (*N. tabacum*, *A. thaliana*, and *S. lycopersicum*). Gray lines represent syntenic blocks between different species, while red lines highlight collinear *RING finger* gene pairs between *L. barbarum* and the compared species. **(D)** Distribution of Ka/Ks ratios. 1: Tandem duplicate *RING finger* genes in *L. barbarum*; 2: Segmental duplicate *RING finger* genes in *L. barbarum*; 3–6: Orthologous *RING finger* genes between *L. barbarum* and *N. tabacum*, *A. thaliana*, and *S. lycopersicum*.

Chromosomal localization analysis revealed an uneven distribution of the 213 *RING finger* genes across the 12 chromosomes of the *L. barbarum* genome. Notably, Chr06 harbored the highest number of genes (30, 14.08%), followed by Chr02 and Chr12, which each contained 21 genes (9.86%). Chr04, Chr08, and Chr10 also exhibited relatively high gene densities, with 20 genes (9.39%) located on each. The remaining chromosomes housed fewer members, specifically Chr01 (18, 8.45%), Chr03 (17, 7.98%), Chr07 (14, 6.57%), Chr11 (11, 5.16%), Chr09 (9, 4.23%), and Chr05 (8, 3.76%). Furthermore, 10 pairs of tandemly duplicated genes were identified, and these duplications were exclusively restricted to four chromosomes: Chr03, Chr06, Chr07, and Chr10 (**Figure 3A**).

Intra-genomic collinearity analysis revealed that 74 *RING finger* genes were involved in 50 segmental duplication pairs. These duplicated pairs were widely distributed across the 12 chromosomes, with the highest frequency observed on Chr01 and the lowest on Chr05 (**Figure 3B**), suggesting that segmental duplication events have played a pivotal role in the expansion of this gene family. To further elucidate the evolutionary history of the *RING finger* family, an inter-species syntenic analysis was performed. The results identified 235, 48, and 13 homologous gene pairs between *L. barbarum* and *S. lycopersicum*, *A. thaliana*, and *N. tabacum*, respectively (**Figure 3C**).

To investigate the evolutionary selection pressures acting on the *L. barbarum RING finger* genes, a Ka/Ks analysis was conducted on both intra-species gene duplication events and inter-species syntenic gene pairs (**Figure 3D**). The Ka/Ks values for all 10 tandemly duplicated gene pairs were less than 1 (**Supplementary Table 2**). Similarly, 46 of the 50 segmentally duplicated pairs exhibited Ka/Ks ratios < 1 (**Supplementary Table 3**), indicating that the vast majority of these duplication events have been subjected to purifying selection. Regarding the inter-species homologous pairs, all 13 pairs between *L. barbarum* and *N. tabacum* displayed Ka/Ks < 1 (**Supplementary Table 4**). Furthermore, 24 of the 48 pairs with *A. thaliana* (**Supplementary Table 5**) and 221 of the 235 pairs with *S. lycopersicum* (**Supplementary Table 6**) were also characteristic of purifying selection. Collectively, these findings suggest that the *L. barbarum RING finger* genes are highly evolutionarily conserved. Notably, Ks and Ka/Ks values for a minor subset of the collinear gene pairs could not be computed, resulting in undefined (‘NaN’) outputs.

### 3.5 Phylogenetic analysis of the *RING finger* gene family in *L. barbarum*

**Figure 4:**
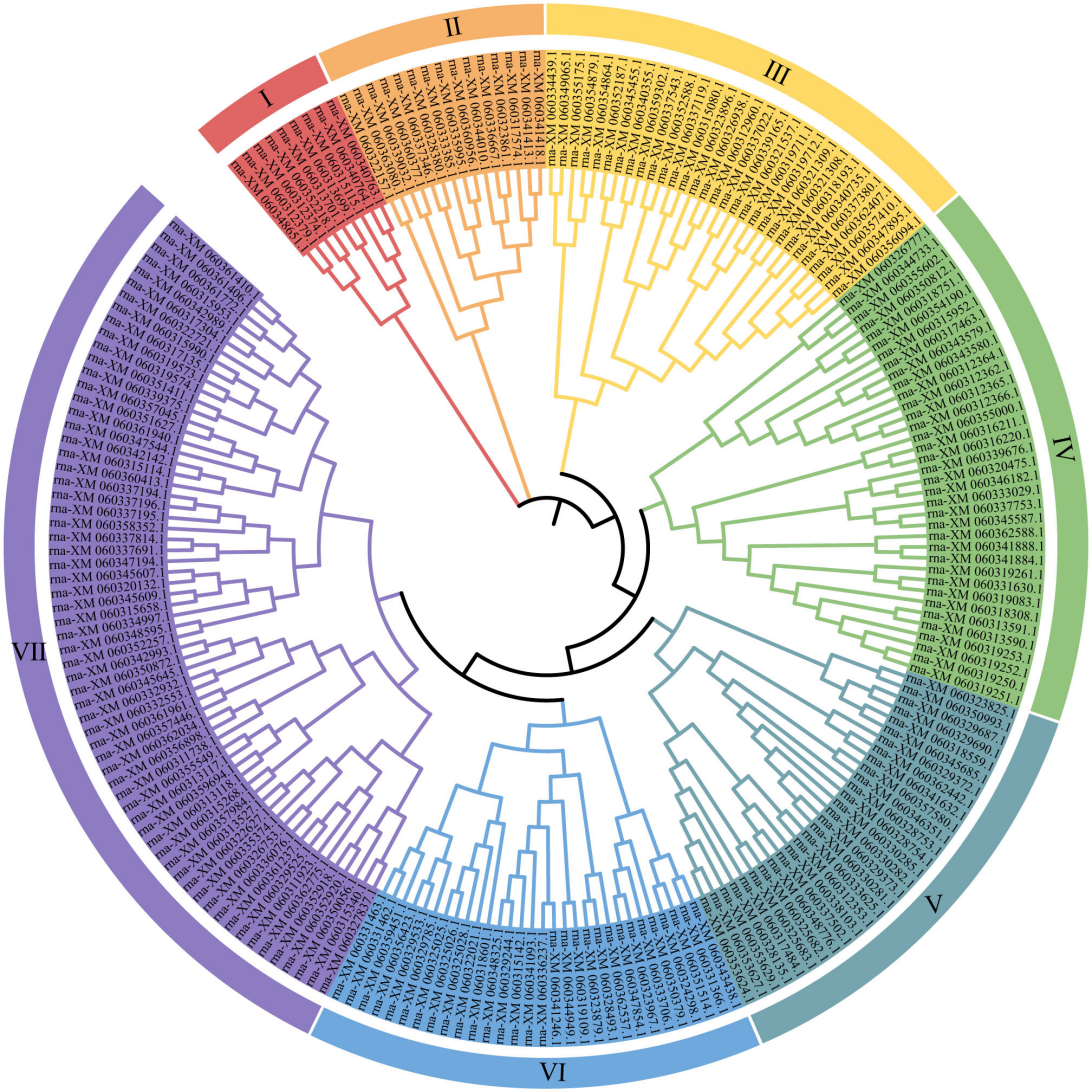
Phylogenetic tree of the *RING finger* gene family in *L. barbarum*. The seven clades (I–VII) are distinguished by different colors. Group names and protein IDs are displayed in the outer ring of the phylogenetic tree.

Phylogenetic analysis clustered the 213 *RING finger* genes into seven distinct subfamilies (**Figure 4**). Among these, Subfamily VII was the largest, comprising 64 members, whereas Subfamily I was the smallest, containing only 9 members. Further inspection revealed that genes encoding identical domain types were not strictly clustered within the same clades, indicating substantial sequence divergence in regions outside the conserved RING domain. Furthermore, the consistent results derived from the analyses of motif composition, domain distribution, and gene structure provided robust support for this seven-subfamily classification, thereby validating the reliability of the phylogenetic clustering.

### 3.6 Prediction of *SBP1* genes in *L. barbarum*

SBP1, a plant-specific RING finger E3 ubiquitin ligase, serves as an essential component of the SCF^SLF/SFB^ ubiquitin ligase complex. Previous studies have demonstrated that SBP1 interacts non-specifically with S-RNase and mediates the recognition and degradation of self-pollen via the ubiquitination pathway, thereby regulating SI (Hua and Kao, 2006). To identify potential SI-related factors in *L. barbarum*, we performed phylogenetic analysis based on SBP1 homologs with established SI-regulatory functions in other plant species, aiming to screen for SI-associated *SBP1* genes in *L. barbarum*.

**Figure 5:**
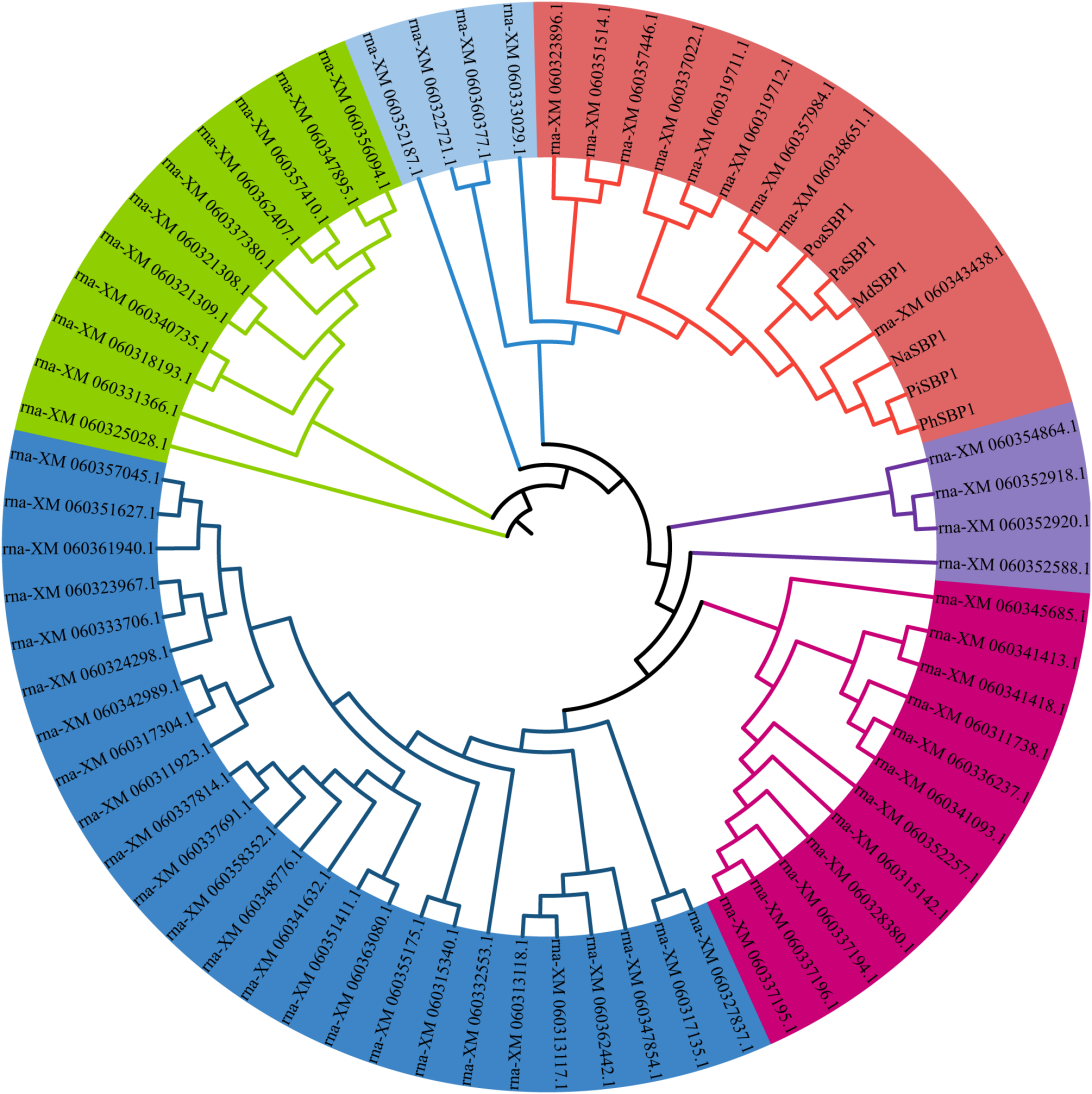
Phylogenetic tree of 65 RING proteins in *L. barbarum* and SBP1 homologs. The SBP1 proteins from other species include MdSBP1, PaSBP1, NaSBP1, PoaSBP1, PhSBP1, and PiSBP1. Clades I–VI are highlighted in different colors. Group names and protein IDs are displayed in the outer ring of the phylogenetic tree.

Within the 213 *L. barbarum RING finger* members, 65 proteins exhibited amino acid lengths ranging from 290–380 residues. Of these, 39 were classified as the RING-H2 type, 25 as the RING-HC type, and a single member as the RING-v type. To further elucidate their evolutionary relationships, a phylogenetic tree comprising these 65 *L. barbarum* RING finger proteins alongside six known SBP1 homologs (MdSBP1, PaSBP1, NaSBP1, PoaSBP1, PhSBP1, and PiSBP1)was constructed. The clustering analysis resolved these proteins into six distinct clades, designated Group I through Group VI (**Figure 5**), which contained 9, 4, 12, 25, 11, and 4 members, respectively. Notably, all six SBP1 homologs were tightly clustered within Group I, and the nine *L. barbarum RING finger* members within this clade exclusively possessed the RING-HC domain.

BLAST analysis revealed that members rna-XM_060357984.1 and rna-XM_060348651.1 shared high homology with SBP (S-ribonuclease binding protein) family proteins from other species. Furthermore, member rna-XM_060343438.1 exhibited significant homology to SBP1 proteins from *N. alata*, *S. chacoense*, and *S. pinnatisectum*, identifying it as an SBP1-type protein in *L. barbarum*, which was subsequently designated *LbSBP1*.

### 3.7 Expression analysis of *RING finger* genes in *L. barbarum*

**Figure 6:**
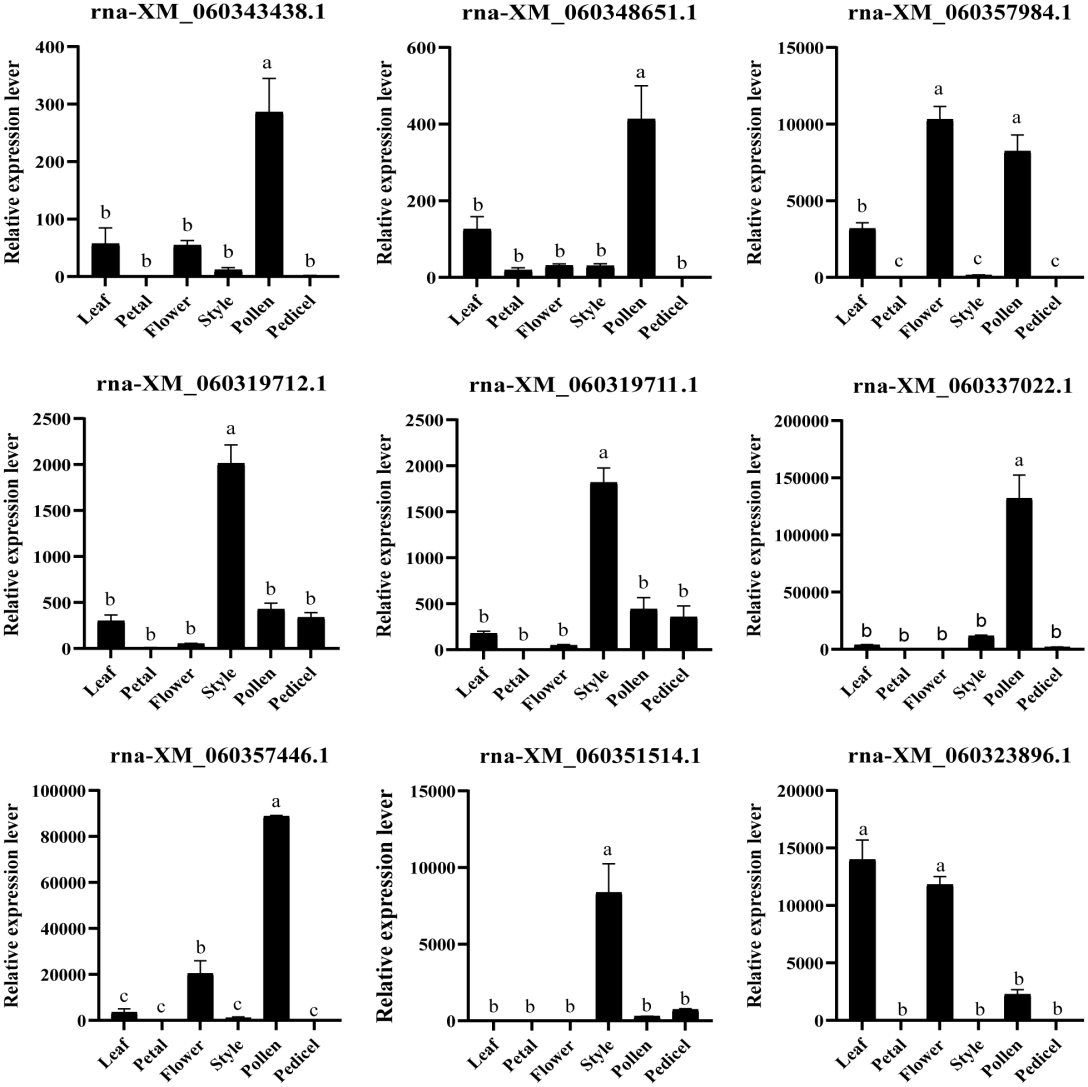
Differential expression analysis of *RING finger* gene members in *L. barbarum*. qRT-PCR analysis was performed to examine the expression levels of nine *RING finger* members in *L. barbarum* that clustered with SBP1 proteins from other species. The tissues analyzed included young leaves, petals, whole flowers, styles, pedicels, and pollen from the ‘Ningqi 1’ cultivar. rna-XM_060343438.1 is identified as the *SBP1* gene in *L. barbarum*. Different lowercase letters above the bars indicate significant differences (P < 0.05).

The expression profiles of the nine genes within Group I were investigated across six distinct tissues: young leaves, petals, whole flowers, styles, pedicels, and pollen (**Figure 6**). The results demonstrated significant tissue-specific expression patterns among these genes. Notably, *LbSBP1* (rna-XM_060343438.1) exhibited significantly higher expression levels in pollen compared to other tissues, while being virtually undetectable in petals and pedicels. Similarly, three additional genes (rna-XM_060348651.1, rna-XM_060337022.1, and rna-XM_060357446.1) were predominantly and highly expressed in pollen, with relatively low transcript abundance in the remaining tissues. Gene rna-XM_060357984.1 displayed elevated expression in both whole flowers and pollen, but was nearly absent in petals, styles, and pedicels. Conversely, the expression levels of rna-XM_060319712.1, rna-XM_060319711.1, and rna-XM_060351514.1 peaked in the style, with comparatively lower abundance in young leaves, petals, and whole flowers. Furthermore, rna-XM_060323896.1 showed relatively high expression in young leaves and whole flowers, but lacked any detectable transcripts in petals, styles, and pedicels. Overall, the majority of genes in Group I displayed a strong tendency for specific expression in either pollen or the style, implying their potential involvement in reproductive processes.

### 3.8 Subcellular localization analysis of *LbSBP1*

**Figure 7:**
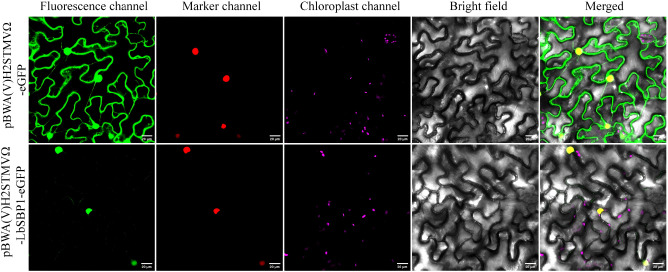
Subcellular localization of *LbSBP1*. The top row displays the empty vector control, and the bottom row shows the localization of *LbSBP1*. For each row, images from left to right represent the fluorescent signal, nuclear marker signal, chloroplast autofluorescence, bright-field, and merged images. Scale bar = 20 μm.

Subcellular localization analysis revealed that in epidermal cells of *N. benthamiana*, the green fluorescent signal in the control group was dispersed throughout the entire cell, whereas in the experimental group, the fluorescence was specifically enriched in the nucleus. This indicates that the LbSBP1 protein predominantly localizes to the nucleus, with a minor fraction distributed in the cytoplasm (**Figure 7**), an observation highly consistent with *in silico* subcellular localization predictions. Furthermore, colocalization assays confirmed that LbSBP1 and the nuclear localization signal marker protein co-localized within the nucleus. This finding corroborates the nuclear localization characteristic of LbSBP1, suggesting that it likely exerts its primary biological functions within the nuclear compartment.

### 3.9 Analysis of the interaction between *SBP1* and *SLF*

**Figure 8:**
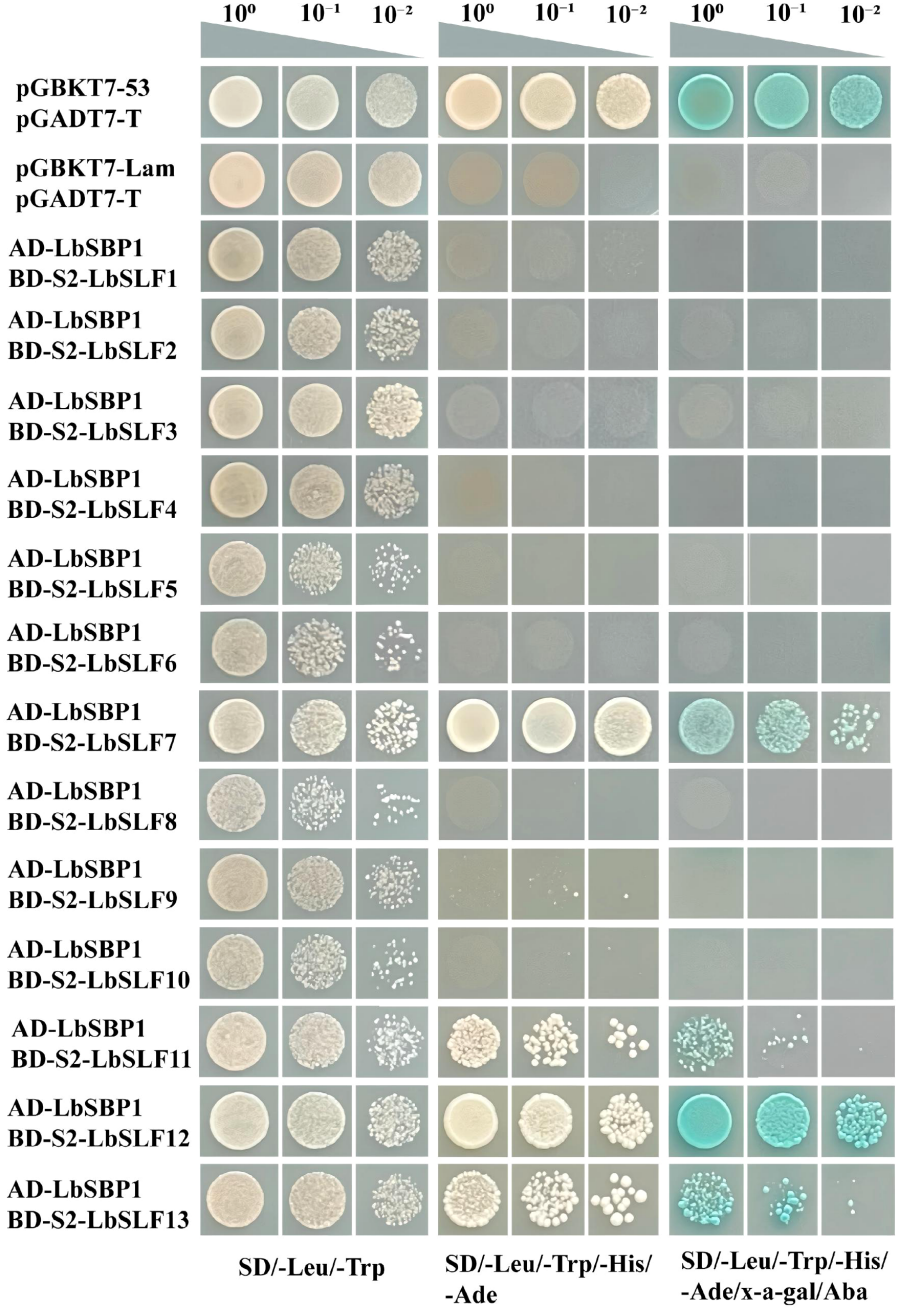
Yeast two-hybrid (Y2H) assay confirming the interaction between SBP1 and SLF. The AD and BD vectors were co-transformed into the *Saccharomyces cerevisiae* Y2HGold strain. Transformants were cultured on DDO (SD/-Leu/-Trp) and QDO/x-a-gal/Aba (SD/-Leu/-Trp/-His/-Ade/X-α-Gal/AbA) media at 30°C for 3–4 days. The yeast cell suspensions were spotted in 10-fold serial dilutions (100, 10-1, 10-2, from left to right). pGBKT7-53 + pGADT7-T served as the positive control, while pGBKT7-Lam + pGADT7-T served as the negative control.

Y2H assays were employed to investigate the protein-protein interactions between LbSBP1 and SLF proteins. The results indicated that the BD vectors fused with *S_2_-LbSLF5*, *S_2_-LbSLF8*, and *S_2_-LbSLF10* exhibited slight autoactivation, as evidenced by sparse colony growth, whereas no autoactivation was observed for the remaining BD-fused constructs (**Supplementary Figure 1A**). Similarly, the AD vector harboring *LbSBP1* showed no colony formation, confirming the absence of autoactivation for this construct (**Supplementary Figure 1B**). On the stringent QDO/x-α-Gal/AbA medium, yeast co-transformed with *LbSBP1* and *S_2_-LbSLF7*, *S_2_-LbSLF11*, *S_2_-LbSLF12*, or *S_2_-LbSLF13* developed robust colonies, confirming positive interactions. Notably, the interaction strength varied, with *S_2_-LbSLF12* exhibiting the strongest binding affinity to *LbSBP1*, followed sequentially by *S_2_-LbSLF7*, *S_2_-LbSLF13*, and *S_2_-LbSLF11* (**Figure 8**). Additionally, while the co-transformation of *LbSBP1* and *S_2_-LbSLF9* yielded a few colonies on the less stringent QDO medium, no growth was detected on the highly stringent QDO/x-α-Gal/AbA plates, indicating that the interaction between *LbSBP1* and *S_2_-LbSLF9* is relatively weak (**Supplementary Figure 1C**).

## 4 Discussion

### 4.1 Identification of 213 *RING finger* genes in *L. barbarum*

RING finger proteins are ubiquitously distributed across eukaryotes, including plants and animals, and represent a major family of E3 ubiquitin ligases (Han et al., 2022). They play pivotal roles in regulating ubiquitination, substrate recognition, and cellular signal transduction (Cho et al., 2017). To date, *RING finger* genes have been systematically identified in various plant species, including *Arabidopsis* (469) (Stone et al., 2005), rice (425) (Lim et al., 2010), *S. lycopersicum* (469) (Yang et al., 2019), poplar (91) (Liu et al., 2015), flax (574) (Meng et al., 2021), apple (663) (Li et al., 2011), cabbage (715) (Alam et al., 2017), and green algae (65) (Gao et al., 2016). In contrast, systematic characterization of the *RING finger* gene family in *L. barbarum* remains largely unexplored. Therefore, we conducted a genome-wide identification of the *RING finger* gene family in *L. barbarum*, yielding a total of 213 members. This number is comparatively lower than that in *S. lycopersicum* and rice, suggesting that the *RING finger* gene family likely underwent lineage-specific divergence during the evolutionary history of different species in response to environmental selective pressures.

Analysis of the physicochemical properties revealed considerable variation in the lengths of the 213 RING finger proteins. Notably, 85.92% of the members were predicted to be hydrophilic, and 61.03% exhibited a pI of less than 7, indicating that the majority of these proteins are inherently unstable and predominantly acidic under physiological conditions. Subcellular localization predictions indicated a predominant nuclear accumulation, suggesting that these proteins primarily exert their functions within the nucleus. This finding aligns well with the physicochemical profiling of RING finger proteins in potato (*S. tuberosum* L.) reported by Chen et al. (2024). Based on domain architecture, these proteins were initially categorized into three subfamilies: RING-H2 (133), RING-HC (64), and RING-v (16). The RING-H2 and RING-HC types constituted a substantial proportion of the gene family, a pattern reminiscent of the identification profiles in rice and *Arabidopsis*; however, the absolute quantities of all three domain types were lower than those observed in *Arabidopsis*. Furthermore, the distribution of specific RING variants—such as RING-C2, RING-S/T, RING-D, and RING-G—exhibits distinct interspecific variation. For instance, the RING-D domain is present in *Arabidopsis* but entirely absent in rice (Jiménez-López et al., 2018). Notably, none of these four domain types were detected in *L. barbarum*, highlighting a unique evolutionary loss or divergence that warrants further comprehensive investigation.

In the present study, cis-acting elements within the 2,000 bp upstream promoter regions of the *L. barbarum RING finger* members were analyzed. A diverse array of elements associated with light responses, phytohormone regulation, abiotic and biotic stresses, and developmental processes was identified. Specifically, elements implicated in MeJA, ABA, SA, GA, and auxin responsiveness, as well as circadian rhythm regulation, meristem specificity, defense and stress responsiveness, and anaerobic induction, were detected. These findings suggest that *RING finger* genes play particularly crucial roles in the growth, development, stress responses, and phytohormone signal transduction of *L. barbarum*, an observation that aligns well with previous reports in *Arabidopsis* and maize (Brugière et al., 2017). Consequently, the characterization of cis-acting elements provides critical clues for further elucidating the functions of individual *RING finger* members. Furthermore, the phylogenetic grouping revealed significant heterogeneity in the amino acid motif compositions, domain types, and gene structures among the *L. barbarum* RING finger proteins, indicating potential functional divergence, a feature analogous to that observed in *S. lycopersicum*. Notably, a strict one-to-one correspondence between protein motifs and domain types was not observed; however, Motif 2 was conserved across nearly all members, being absent in only a few genes, which implies a fundamental role for this motif within the gene family. Structural analysis demonstrated that genes harboring the RING-H2, RING-HC, and RING-v domains possessed 1 to 13, 1 to 19, and 2 to 9 exons, respectively. Notably, genes clustered within the same clade of the phylogenetic tree exhibited high degrees of consistency in their exon-intron architectures (Zhao et al., 2024). This topological similarity suggests that the gene family has been subjected to strong evolutionary constraints, thereby maintaining the structural conservation of these genes. Taken together, our findings indicate that the *L. barbarum* RING finger protein family has evolved distinct molecular evolutionary patterns over its long-term evolutionary history, ultimately enabling the precise regulation of the *L. barbarum* life cycle.

The 213 *L. barbarum RING finger* genes thus uncovered were mapped onto 12 chromosomes with uneven distribution densities, a pattern consistent with the findings of Yang et al. in *S. lycopersicum*. Genomic duplication events serve as major drivers of gene family expansion. Segmental and tandem duplications represent the two primary mechanisms underlying the expansion of plant gene families, playing pivotal roles in genetic functional innovation and the formation of multi-gene families (Yang et al., 2019; Wang et al., 2025). In the present study, we identified 10 tandem duplication events and 50 segmental duplication events among the *L. barbarum RING finger* members, indicating that both mechanisms synergistically drove the expansion of this gene family. Comparatively, *S. lycopersicum* possesses 149 segmental and 49 tandem duplicated gene pairs, underscoring the critical significance of duplication events in amplifying the *RING finger* gene repertoire within the Solanaceae family. Synteny analysis provides critical insights into gene evolutionary trajectories. Interspecific synteny analysis identified 48, 235, and 13 collinear gene pairs between *L. barbarum* and *A. thaliana*, *S. lycopersicum*, and *N. tabacum*, respectively. This extensive collinear network suggests that the *RING finger* genes in these species likely originated from a common ancestor, subsequently undergoing varying degrees of duplication and divergence during their evolutionary histories. These findings offer a theoretical framework for elucidating the genetic evolutionary mechanisms of this gene family across different species. Furthermore, the Ka/Ks ratio is a widely utilized metric for assessing the direction and intensity of natural selection pressures acting on protein-coding genes (Hurst, 2002). In the *L. barbarum* genome, the vast majority of homologous gene pairs exhibited a Ka/Ks ratio of < 1, indicating that these *RING finger* genes have been subjected to purifying selection, thereby maintaining a high degree of functional conservation. However, purifying selection does not necessarily preclude functional divergence. Under strong selective constraint, duplicated *RING finger* genes may still undergo sub functionalization, whereby the ancestral function is partitioned into distinct spatiotemporal expression domains without altering coding sequences (Lynch and Force, 2000; Birchler, 2025). Thus, although no neofunctionalization signal (Ka/Ks > 1) was detected in this family, the possibility of sub functionalization remains open and requires further validation through expression profiling or complementation assays. Notably, the Ka/Ks and Ks values for a minor subset of sequence pairs could not be calculated due to insufficient evolutionary divergence. This phenomenon likely stems either from an extremely short divergence time or exceptionally strong purifying selection, where the mutation level falls below the reliable detection threshold of the computational model. Consequently, these specific genes are hypothesized to be of very recent origin or to possess ultra-conserved characteristics. Beyond the aforementioned insufficient divergence, additional biological mechanisms may also lead to non-computable “NaN” results. These include the presence of premature stop codons indicative of pseudogenization (Khachane and Harrison, 2009), extremely short alignment lengths that preclude reliable estimation, and uniform selective constraints lacking site-to-site heterogeneity, which can cause the posterior distribution of dN–dS to lie entirely on one side of zero (Pond and Muse, 2005). Hence, these NaN cases are not merely technical artifacts but may reflect genuine evolutionary events such as incipient functional decay, very recent duplication, or lineage-specific ultra-conservation.

### 4.2 LbSBP1: one of the *RING finger* members in *Lycium barbarum*

Based on the phylogenetic tree, the 213 *RING finger* members were classified into seven subfamilies (I–VII), exhibiting considerable variation in member abundance among the clades. Notably, members harboring identical domain types did not form tight clusters, indicating substantial sequence divergence outside the RING domain regions. This observation aligns with the clustering patterns of *RING finger* members previously reported in *Arabidopsis* and *S. lycopersicum*. From this gene repertoire, a specific member containing a RING-HC domain was identified as *L. barbarum* SBP1 (*LbSBP1*), laying a foundation for subsequent functional characterization. Previous studies have established that SBP1 represents a class of RING finger proteins that act as key factors in S-RNase-based SI reactions in plants (Sims and Ordanic, 2001). In *P. inflata* and *P. tenella*, SBP1 functionally mimics SSK1 (Skp1-like) and Rbx1 to assemble an atypical SCF^SLF/SFB^ complex containing SBP1, which ubiquitinates non-self S-RNases, thereby regulating GSI (Zeng et al., 2019; Hua and Kao, 2006). Notably, SBP1 homologs exhibit broad expression profiles beyond SI responses. For instance, O’Brien et al. (2004) identified *ScSBP1* in potato, with expression detected in most examined tissues except tubers, implying its involvement in diverse biological processes. Similarly, Lee et al. (2008) reported that *NaSBP1* in *N. alata* is expressed in pollen and persists across various developmental stages of pistils and pollen (from buds to mature flowers). Furthermore, Minamikawa et al. (2013) identified *MdSBP1* in apple pollen; RT-PCR analysis confirmed its constitutive expression across all analyzed organs, suggesting roles extending beyond pollination to other fundamental cellular functions. In the present study, the transcript XM_060343438.1 was identified as *LbSBP1*. Expression analysis revealed its most prominent abundance in pollen, alongside detectable expression in young leaves, flowers, and styles. This multi-tissue expression pattern implies a role in pollen growth and development, which corresponds with the broad expression profiles of *ScSBP1* in potato and *MdSBP1* in apple. Subcellular localization prediction indicated that LbSBP1 is predominantly localized in the nucleus. This spatial distribution is consistent with the localization of *A. thaliana* AtSBP1 and various other RING-HC-containing proteins (e.g., COP1 and DRIP1) in *A. thaliana* (Valassakis et al., 2019; Deng et al., 1992; Qin et al., 2008). In conclusion, while the present study successfully identified and preliminarily characterized *LbSBP1*, critical questions remain. Future investigations are required to elucidate whether LbSBP1 functionally substitutes for SSK1 and Rbx1 to assemble the SCF^SLF/SFB^ complex, how it mediates pollen-specific functions, and to what extent it participates in the regulation of SI in *L. barbarum*.

### 4.3 Interaction of LbSBP1 with SI pollen determinants LbSLFs

SI is a reproductive isolation mechanism in flowering plants that prevents self-fertilization by utilizing the genetic diversity of the S-locus to distinguish between self and non-self-pollen (Muñoz-Sanz et al., 2020). Pollen-derived SLF proteins represent a class of typical F-box proteins that incorporate into the SCF complex to specifically recognize non-self S-RNases, targeting them for ubiquitination and subsequent degradation. Conversely, self S-RNases escape degradation, thereby inhibiting pollen tube growth and culminating in the SI response (Sijacic et al., 2004; Weissman, 2001). SBP1 (S-RNase Binding Protein 1), acting as a conserved Skp1-like protein, directly interacts with SLFs, mediating their integration into the SCF complex to facilitate specific recognition (Huang et al., 2006). In *P. inflata*, PiSBP1 was found to interact with S2-SLF1 and PiCUL1-G, whereas PiSSK1 failed to interact with either component. This led to the hypothesis that within the *P. inflata* SI system, PiSBP1 functionally substitutes for PiSSK1 to bridge the F-box protein and CUL1, thereby assembling the SCF complex (Hua and Kao, 2006). Similarly, in *P. tenella*, interactions were detected between PetSBP1 and PetCUL1, as well as PetSFBs (SFB16 and SFB17), leading to the proposition that a novel SBP1-containing SCF complex exists in this species (Zeng et al., 2019). Conversely, studies in apple (*M. domestica*) revealed that MdSBP1 lacks binding affinity for MdCUL1, MdSFBB, and MdSFBL, indicating that MdSBP1 does not associate with CUL1 and SFB to form an SCF complex in this context (Yuan et al., 2014). In the present study, Y2H assays demonstrated that *L. barbarum LbSBP1* physically interacts with *S_2_-LbSLF7*, *S_2_-LbSLF9*, *S_2_-LbSLF11*, *S_2_-LbSLF12*, and *S_2_-LbSLF13*. This suggests that *LbSBP1* may be involved in the assembly of an atypical SCF complex, thereby modulating the SI response in *L. barbarum*. However, whether *LbSBP1* interacts with *LbCUL1* remains to be elucidated; thus, the *in vivo* existence of an LbSBP1-incorporating SCF complex requires further experimental validation.

## 5 Conclusion

In this study, we identified 213 *RING finger* genes in the *L. barbarum* genome. These genes were classified into seven subfamilies and widely distributed across 12 chromosomes, with phylogenetic analysis categorizing them into three distinct types: RING-H2, RING-HC, and RING-v. Notably, a specific member of this family, *LbSBP1*, was characterized; this gene exhibits pollen-specific expression and localizes to the nucleus. Furthermore, *LbSBP1* was found to interact with the SI pollen determinants in *L. barbarum*, specifically *S_2_-LbSLF7*, *S_2_-LbSLF9*, *S_2_-LbSLF11*, *S_2_-LbSLF12*, and *S_2_-LbSLF13*. Collectively, these findings enrich our current understanding of the *RING finger* gene family and provide a theoretical basis for future studies, with *LbSBP1* representing a candidate gene whose potential role in self-incompatibility warrants further investigation.

## Data Availability

The original contributions presented in the study are included in the article/**Supplementary Material**. Further inquiries can be directed to the corresponding author.

## References

[B1] Alam I. Yang Y. Q. Wang Y. Zhu M. L. Wang H. B. Chalhoub B. . (2017). Genome-wide identification, evolution and expression analysis of RING finger protein genes in Brassica rapa. Sci. Rep. 7, 40690. doi: 10.1038/srep40690 28094809 PMC5240574

[B2] Bailey T. L. Mikael B. Buske F. A. Martin F. Grant C. E. Luca C. . (2009). MEME SUITE: tools for motif discovery and searching. Nucleic Acids Res. 37, W202–W208. doi: 10.1093/nar/gkp335 19458158 PMC2703892

[B3] Birchler J. A. (2025). When is it subfunctionalization and when is it not? G3 Genes/Genomes/Genetics 15, jkae269. doi: 10.1093/g3journal/jkae269 39699176 PMC11708216

[B4] Brillada C. Trujillo M. (2022). E2 ubiquitin-conjugating enzymes (UBCs): drivers of ubiquitin signalling in plants. Essays Biochem. 66, 99–110. doi: 10.1042/ebc20210093 35766526

[B5] Brugière N. Zhang W. J. Xu Q. Z. Scolaro E. J. Lu C. Kahsay R. Y. . (2017). Overexpression of RING Domain E3 Ligase ZmXerico1 confers drought tolerance through regulation of ABA homeostasis. Plant Physiol. 175, 1350–1369. doi: 10.1104/pp.17.01072 28899960 PMC5664481

[B6] Chen C. J. Chen H. Zhang Y. Thomas H. R. Frank M. H. He Y. H. . (2020). TBtools: an integrative toolkit developed for interactive analyses of big biological data. Mol. Plant 13, 1194–1202. doi: 10.1016/j.molp.2020.06.009 32585190

[B7] Chen L. M. Li Y. M. Zhu J. Y. Li Z. T. Wang W. L. Qi Z. Y. . (2024). Comprehensive characterization of the C3HC4 RING finger gene family in potato (Solanum tuberosum L.): Insights into their involvement in anthocyanin biosynthesis. Int. J. Mol. Sci. 25, 2082. doi: 10.3390/ijms25042082 38396758 PMC10889778

[B8] Chen Y. H. Yang X. Y. He K. Liu M. H. Li J. G. Gao Z. F. . (2006). The MYB transcription factor superfamily of Arabidopsis: expression analysis and phylogenetic comparison with the rice MYB family. Plant Mol. Biol. 60, 107–124. doi: 10.1007/s11103-005-2910-y 16463103

[B9] Cho S. K. Ryu M. Y. Kim J. H. Hong J. S. Oh T. R. Kim W. T. . (2017). RING E3 ligases: key regulatory elements are involved in abiotic stress responses in plants. BMB Rep. 50, 393–400. doi: 10.5483/BMBRep.2017.50.8.128 28712388 PMC5595168

[B10] Ciechanover A. (2015). The unravelling of the ubiquitin system. Nat. Rev. Mol. Cell Biol. 16, 322–324. doi: 10.1038/nrm3982 25907614

[B11] de Heiden R. A. Mulder M. P. C. (2025). Drug ubiquitination: an unwelcome mode of action or a novel modality. Trends Biochem. Sci. 50, 1045–1046. doi: 10.1016/j.tibs.2025.10.004 41162202

[B12] Deng X. W. Matsui M. Wei N. Wagner D. Chu A. M. Feldmann K. A. . (1992). COP1, an Arabidopsis regulatory gene, encodes a protein with both a zinc-binding motif and a G beta homologous domain. Cell. 71, 791–801. doi: 10.1016/0092-8674(92)90555-q 1423630

[B13] Entani T. Kubo K. Isogai S. Fukao Y. Shirakawa M. Isogai A. . (2014). Ubiquitin-proteasome-mediated degradation of S-RNase in a solanaceous cross-compatibility reaction. Plant J. 78, 1014–1021. doi: 10.1111/tpj.12528 24689760

[B14] Fang F. Y. Zhou W. L. Liu Y. F. Song Z. Z. Zheng S. F. Wang F. . (2023). Characterization of RING-type ubiquitin SINA E3 ligases and their responsive expression to salt and osmotic stresses in Brassica napus. Plant Cell Rep. 42, 859–877. doi: 10.1007/s00299-023-02996-w 36788135

[B15] Feng H. J. Li C. Zhou J. L. Yuan Y. Feng Z. L. Shi Y. Q. . (2021). A cotton WAKL protein interacted with a DnaJ protein and was involved in defense against Verticillium dahliae. Int. J. Biol. Macromol. 167, 633–643. doi: 10.1016/j.ijbiomac.2020.11.191 33275973

[B16] Gao Y. Li M. Y. Zhao J. Zhang Y. C. Xie Q. J. Chen D. H. (2016). Genome-wide analysis of RING finger proteins in the smallest free-living photosynthetic eukaryote Ostreococus tauri. Mar. Genomics 26, 51–61. doi: 10.1016/j.margen.2015.12.008 26751716

[B17] Gao Y. M. Liu H. L. Wang Y. J. Li F. Xiang Y. (2018). Genome-wide identification of PHD-finger genes and expression pattern analysis under various treatments in moso bamboo (Phyllostachys edulis). Plant Physiol. Biochem. 123, 378–391. doi: 10.1016/j.plaphy.2017.12.034 29304483

[B18] Han G. L. Qiao Z. Q. Li Y. X. Yang Z. R. Wang C. F. Zhang Y. Y. . (2022). RING zinc finger proteins in plant abiotic stress tolerance. Front. Plant Sci. 13, 877011. doi: 10.3389/fpls.2022.877011 35498666 PMC9047180

[B19] Hu J. B. Du Z. Z. Liu C. C. Wen H. Liu C. Chen P. . (2025). Pan-S-locus analysis reveals insights into the origin and evolution of self-incompatibility in the orange subfamily. Genome Biol. 26, 344. doi: 10.1186/s13059-025-03817-x 41057937 PMC12506349

[B20] Hua Z. Kao T. H. (2006). Identification and characterization of components of a putative Petunia S-locus F-box–containing E3 ligase complex involved in S-RNase–based self-incompatibility. Plant Cell 18, 2531–2553. doi: 10.1105/tpc.106.041061 17028207 PMC1626602

[B21] Huang J. Zhao L. Yang Q. Xue Y. (2006). AhSSK1, a novel SKP1-like protein that interacts with the S-locus F-box protein SLF. Plant J. 46, 780–793. doi: 10.1111/j.1365-313X.2006.02735.x 16709194

[B22] Hurst L. D. (2002). The Ka/Ks ratio: diagnosing the form of sequence evolution. Trends Genet. 18, 486–487. doi: 10.1016/S0168-9525(02)02722-1 12175810

[B23] Ji R. Chen Y. L. Qian C. M. Jin J. P. (2023). Functions of phosphorylated ubiquitin. Prog. Biochem. Biophys. 50, 740–748. doi: 10.16476/j.pibb.2023.0079

[B24] Jiménez-López D. Muñóz-Belman F. González-Prieto J. M. Aguilar-Hernández V. Guzmán P. (2018). Repertoire of plant RING E3 ubiquitin ligases revisited: new groups counting gene families and single genes. PloS One 13, e0203442. doi: 10.1371/journal.pone.0203442 30169501 PMC6118397

[B25] Khachane A. N. Harrison P. M. (2009). Strong association between pseudogenization mechanisms and gene sequence length. Biol. Direct 4, 38. doi: 10.1186/1745-6150-4-38 19807910 PMC2768697

[B26] Kubo K. Entani T. Takara A. Wang N. Fields A. M. Hua Z. H. . (2010). Collaborative non-self recognition system in S-RNase-based self-incompatibility. Science 330, 796–803. doi: 10.1126/science.1195243 21051632

[B27] Lee H. S. Huang S. Kao T. H. (1994). S proteins control rejection of incompatible pollen in Petunia inflata. Nature 367, 560–563. doi: 10.1038/367560a0 7509041

[B28] Lee C. B. Swatek K. N. McClure B. (2008). Pollen proteins bind to the C-terminal domain of Nicotiana alata pistil arabinogalactan proteins. J. Biol. Chem. 283, 26965–26973. doi: 10.1074/jbc.M804410200 18678868

[B29] Letunic I. Bork P. (2019). Interactive Tree Of Life (iTOL) v4: recent updates and new developments. Nucleic Acids Res. 47, W256–W259. doi: 10.1093/nar/gkz239 30931475 PMC6602468

[B30] Letunic I. Khedkar S. Bork P. (2021). SMART: recent updates, new developments and status in 2020. Nucleic Acids Res. 49, D458–D460. doi: 10.1093/nar/gkaa937 33104802 PMC7778883

[B31] Li W. L. Sun Q. Li W. C. Yu Y. L. Zhao M. Meng Z. D. (2017). Characterization and expression analysis of a novel RING-HC gene, ZmRHCP1, involved in brace root development and abiotic stress responses in maize. J. Integr. Agric. 16, 1892–1899. doi: 10.1016/s2095-3119(16)61576-9

[B32] Li Y. Z. Wu B. J. Yu Y. L. Yang G. D. Wu C. G. Zheng C. C. (2011). Genome-wide analysis of the RING finger gene family in apple. Mol. Genet. Genomics 286, 81–94. doi: 10.1007/s00438-011-0625-0 21630098

[B33] Lim S. D. Yim W. C. Moon J. C. Kim D. S. Lee B. M. Jang C. S. (2010). A gene family encoding RING finger proteins in rice: their expansion, expression diversity, and co-expressed genes. Plant Mol. Biol. 72, 369–380. doi: 10.1007/s11103-009-9576-9 19957018

[B34] Liu P. D. Cai Z. F. Chen Z. J. Mo X. H. Ding X. P. Liang C. Y. . (2018). A root-associated purple acid phosphatase, SgPAP23, mediates extracellular phytate-P utilization in Stylosanthes guianensis. Plant Cell Environ. 41, 2821–2834. doi: 10.1111/pce.13412 30066375

[B35] Liu H. Li N. Wang Y. Cheng T. Yang H. Peng Q. (2024). Study on fermentation kinetics, antioxidant activity and flavor characteristics of Lactobacillus plantarum CCFM1050 fermented wolfberry pulp. Food. Innov. Adv. 3, 126–134. doi: 10.48130/fia-0024-0012

[B36] Liu Q. G. Yang J. L. Wang Z. C. Xu X. M. Mao X. L. Li D. D. . (2015). Genome-wide classification, identification and expression profile of the C3HC4-type RING finger gene family in poplar (Populus trichocarpa). Plant Mol. Biol. Rep. 33, 1740–1754. doi: 10.1007/s11105-015-0870-1 30311153

[B37] Liu Z. C. Zhang P. Li M. J. Limayan A. Yang G. H. Yu Y. . (2022). Mrz1, a novel mitochondrial outer membrane RING finger protein, is degraded through the ubiquitin-proteasome pathway in Schizosaccharomyces pombe. Curr. Microbiol. 79, 02298. doi: 10.1007/s00284-022-02998-z 36088506

[B38] Liu Y. C. Zhang W. Y. Li Y. J. Shen R. H. Wang S. B. Wang X. . (2025). Analysis on the difference of nutrient components of black wolfberry seed grown in different regions. Food Res. Int. 221, 117061. doi: 10.1016/j.foodres.2025.117061 41606905

[B39] Livak K. J. Schmittgen T. D. (2001). Analysis of relative gene expression data using real-time quantitative PCR and the 2^-ΔΔCT^ method. Methods 25, 402–408. doi: 10.1006/meth.2001.1262 11846609

[B40] Lorick K. L. Jensen J. P. Fang S. Ong A. M. Hatakeyama S. Weissman A. M. (1999). RING fingers mediate ubiquitin-conjugating enzyme (E2)-dependent ubiquitination. Proc. Natl. Acad. Sci. U.S.A. 96, 11364–11373. doi: 10.1073/pnas.96.20.11364 10500182 PMC18039

[B41] Lynch M. Force A. (2000). The probability of duplicate gene preservation by subfunctionalization. Genetics 154, 459–473. doi: 10.1093/genetics/154.1.459 10629003 PMC1460895

[B42] Ma Y. P. Reddy V. R. Devi M. J. Song L. H. Cao B. (2019). De novo characterization of the Goji berry (Lycium barbarium L.) fruit transcriptome and analysis of candidate genes involved in sugar metabolism under different CO2 concentrations. Tree Physiol. 39, 1032–1045. doi: 10.1093/treephys/tpz014 30824924

[B43] Meng X. W. Liu J. Zhao M. D. (2021). Genome-wide identification of RING finger genes in flax (Linum usitatissimum) and analyses of their evolution. PeerJ 9, e12491. doi: 10.7717/peerj.12491 34820204 PMC8601054

[B44] Minamikawa M. F. Fujii D. Kakui H. Kotoda N. Sassa H. (2013). Identification of an S-RNase binding protein1 (SBP1) homolog of apple (Malus × domestica). Plant Biotechnol. 30, 119–123. doi: 10.5511/plantbiotechnology.13.0109a

[B45] Muñoz-Sanz J. V. Zuriaga E. Cruz-García F. McClure B. Romero C. (2020). Self-(in)compatibility systems: target traits for crop-production, plant breeding, and biotechnology. Front. Plant Sci. 11, 195. doi: 10.3389/fpls.2020.00195 32265945 PMC7098457

[B46] Nettancourt D. D. (1997). Incompatibility in angiosperms. Sex Plant Reprod. 10, 185–197. doi: 10.1007/s004970050087 30311153

[B47] O’Brien M. Major G. Chantha S. C. Matton D. P. (2004). Isolation of S-RNase binding proteins from Solanum chacoense: identification of an SBP1 (RING finger protein) orthologue. Sex Plant Reprod. 17, 81–87. doi: 10.1007/s00497-004-0218-8 30311153

[B48] Peng Y. H. Liu S. Cui L. Liu J. K. Long J. G. (2023). Opportunities and challenges in targeting ubiquitin modification and degradation for prostate cancer therapy. Prog. Biochem. Biophys. 50, 782–794. doi: 10.16476/j.pibb.2022.0568

[B49] Pond S. L. K. Muse S. V. (2005). “ HyPhy: hypothesis testing using phylogenies,” in Statistical Methods in Molecular Evolution ( Springer New York, New York, NY), 125–181. doi: 10.1007/0-387-27733-1_6

[B50] Qin F. Sakuma Y. Tran L.-S. P. Maruyama K. Kidokoro S. Fujita Y. . (2008). Arabidopsis DREB2A-interacting proteins function as RING E3 ligases and negatively regulate plant drought stress–responsive gene expression. Plant Cell 20, 1693–1707. doi: 10.1105/tpc.107.057380 18552202 PMC2483357

[B51] Sijacic P. Wang X. Skirpan A. L. Wang Y. Dowd P. E. McCubbin A. G. . (2004). Identification of the pollen determinant of S-RNase-mediated self-incompatibility. Nature 429, 302–305. doi: 10.1038/nature02523 15152253

[B52] Sims T. L. Ordanic M. (2001). Identification of a S-ribonuclease-binding protein in Petunia hybrida. Plant Mol. Biol. 47, 771–783. doi: 10.1023/A:1013639528858 11785938

[B53] Stone S. L. Hauksdóttir H. Troy A. Herschleb J. Kraft E. Callis J. (2005). Functional analysis of the RING-type ubiquitin ligase family of Arabidopsis. Plant Physiol. 137, 13–30. doi: 10.1104/pp.104.052423 15644464 PMC548835

[B54] Tamura K. Stecher G. Kumar S. (2021). MEGA11 molecular evolutionary genetics analysis version 11. Mol. Biol. Evol. 38, 3022–3027. doi: 10.1093/molbev/msab120 33892491 PMC8233496

[B55] Tian H. Y. Zhang H. K. Huang H. Q. Zhang Y. E. Xue Y. B. (2024). Phase separation of S-RNase promotes self-incompatibility in Petunia hybrida. J. Integr. Plant Biol. 66, 986–1006. doi: 10.1111/jipb.13584 37963073

[B56] Valassakis C. Dervisi I. Agalou A. Papandreou N. Kapetsis G. Podia V. . (2019). Novel interactions of Selenium Binding Protein family with the PICOT containing proteins AtGRXS14 and AtGRXS16 in Arabidopsis thaliana. Plant Sci. 281, 102–112. doi: 10.1016/j.plantsci.2019.01.021 30824043

[B57] Vekemans X. Castric V. (2021). When the genetic architecture matters: evolutionary and ecological implications of self versus nonself recognition in plant self-incompatibility Comment. New Phytol. 231, 1304–1307. doi: 10.1111/nph.17471 34146416

[B58] Wang X. Q. Chen E. Y. Ge X. Y. Gong Q. Butt H. Zhang C. J. . (2018). Overexpressed BRH1, a RING finger gene, alters rosette leaf shape in Arabidopsis thaliana. Sci. China Life Sci. 61, 79–87. doi: 10.1007/s11427-017-9133-8 28887625

[B59] Wang Q. Guo C. Li Z. Y. Sun J. H. Deng Z. C. Wen L. C. . (2021). Potato NAC transcription factor StNAC053 enhances salt and drought tolerance in transgenic Arabidopsis. Int. J. Mol. Sci. 22, 2568. doi: 10.3390/ijms22052568 33806406 PMC7961516

[B60] Wang C. P. Qin K. Shang X. H. Gao Y. Wu J. L. Ma H. J. . (2024). Mapping quantitative trait loci associated with self-(in)compatibility in goji berries (Lycium barbarum). BMC Plant Biol. 24, 441. doi: 10.1186/s12870-024-05092-7 38778301 PMC11112781

[B61] Wang Y. Wang H. Li W. Dai G. Chen J. (2025). Genome-wide identification and expression analysis of the LbDof transcription factor family genes in Lycium barbarum. Plants 14, 1567. doi: 10.3390/plants14111567 40508247 PMC12157807

[B62] Wang C. L. Zhu M. Hong H. Li J. Zuo C. K. Zhang Y. . (2024). A viral effector blocks the turnover of a plant NLR receptor to trigger a robust immune response. EMBO J. 43, 3650–3676. doi: 10.1038/s44318-024-00174-6 39020150 PMC11377725

[B63] Weissman A. M. (2001). Themes and variations on ubiquitylation. Nat. Rev. Mol. Cell Biol. 2, 169–178. doi: 10.1038/35056563 11265246

[B64] Wilkins M. R. Gasteiger E. Bairoch A. Sanchez J. C. Hochstrasser D. F. (1999). Protein identification and analysis tools in the ExPASy server. Methods Mol. Biol. 112, 531–552. doi: 10.1385/1-59259-890-0:571 10027275

[B65] Wu J. L. Nan X. X. Qin K. Dai G. L. Zhang X. Yang Z. J. . (2025). The cytological basis of self-incompatibility in goji (Lycium barbarum) and the cloning of S-RNase gene. Planta 262, 33. doi: 10.1007/s00425-025-04753-7 40536558

[B66] Xue Y. B. (2025). S-RNase-based self-incompatibility in angiosperms: Degradation, condensation, and evolution. Plant Physiol. 199, kiaf360. doi: 10.1093/plphys/kiaf360 40811654 PMC12403065

[B67] Yan S. S. Wang Y. X. Yu B. W. Gan Y. W. Lei J. J. Chen C. M. . (2024). A putative E3 ubiquitin ligase substrate receptor degrades transcription factor SmNAC to enhance bacterial wilt resistance in eggplant. Hortic. Res. 11, uhad246. doi: 10.1093/hr/uhad246 38239808 PMC10794948

[B68] Yang M. Derbyshire M. K. Yamashita R. A. Marchler-Bauer A. (2020). NCBI's conserved domain database and tools for protein domain analysis. Curr. Protoc. Bioinf. 69, e90. doi: 10.1002/cpbi.90 31851420 PMC7378889

[B69] Yang L. Miao M. J. Lyu H. J. Cao X. Li J. Li Y. J. . (2019). Genome-wide identification, evolution, and expression analysis of RING finger gene family in Solanum lycopersicum. Int. J. Mol. Sci. 20, 4864. doi: 10.3390/ijms20194864 31574992 PMC6801689

[B70] Yuan H. Meng D. Gu Z. Y. Li W. Wang A. D. Yang Q. . (2014). A novel gene, MdSSK1, as a component of the SCF complex rather than MdSBP1 can mediate the ubiquitination of S-RNase in apple. J. Exp. Bot. 65, 3121–3131. doi: 10.1093/jxb/eru164 24759884 PMC4071834

[B71] Zeng B. Wang J. Y. Hao Q. Yu Z. F. Abudukayoumu A. Tang Y. L. . (2019). Identification of a novel SBP1-containing SCF^SFB^ complex in wild dwarf almond (Prunus tenella). Front. Genet. 10, 1019. doi: 10.3389/fgene.2019.01019 31708966 PMC6823244

[B72] Zhang X. Ma X. M. Yang G. Yin X. Yao W. K. Tigabu M. . (2025). Establishment of an efficient regeneration system for Lycium barbarum. Ind. Crops Prod. 226, 120698. doi: 10.1016/j.indcrop.2025.120698 38826717

[B73] Zhang R. R. Wu Y. Qu X. R. Yang W. J. Wu Q. Huang L. . (2024). The RING-finger ubiquitin E3 ligase TaPIR1 targets TaHRP1 for degradation to suppress chloroplast function. Nat. Commun. 15, 51249. doi: 10.1038/s41467-024-51249-1 39134523 PMC11319775

[B74] Zhao J. Li A. Xu M. Dai G. Chen J. (2024). Genome-wide analysis of the TIFY family in Lycium and the negative regulation of stomatal development by LrJAZ2 gene. Plant Physiol. Biochem. 206, 108285. doi: 10.1016/j.plaphy.2023.108285 38145586

