# Supplementary material for: Genome-wide identification and analysis of RING finger gene family and the self-compatibility-associated SBP1 gene in goji berry (Lycium barbarum)
Source: Front Plant Sci. 2026 Jun 30;17:1863389. doi: 10.3389/fpls.2026.1863389 (PMC13365326; doi:10.3389/fpls.2026.1863389)
Supplement: Supplementary file 1 [file DataSheet1.zip › Supplementary Materials/Supplementary Material 7/Supplementary Fig. 1.docx]

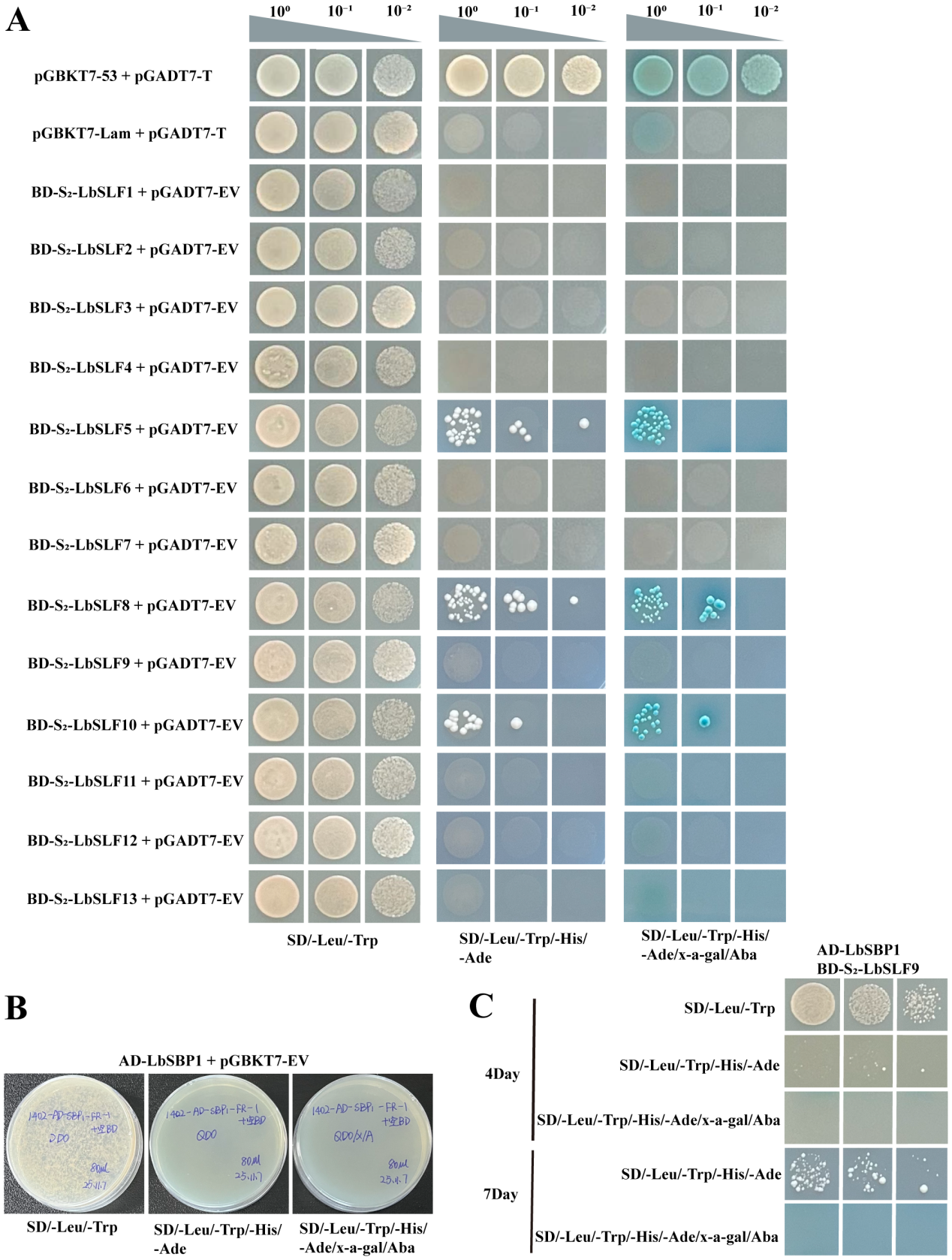


**Supplementary Fig. 1** Self-activation assays of *S_2_-LbSLFs* and *LbSBP1*, and weak interaction verification of *LbSBP1*/*S_2_-LbSLF9*.

The full-length sequences of *LbSBP1* and 13 *SLF* genes were co-transformed in pairs, and their growth phenotypes were assessed on selective media: DDO (SD/-Leu/-Trp), QDO (SD/-Leu/-Trp/-His/-Ade), and QDO/X-α-Gal/AbA (SD/-Leu/-Trp/-His/-Ade/X-α-Gal/AbA). All co-transformed combinations exhibited normal growth on DDO plates, confirming the efficiency of the transformation and inoculation procedures. On QDO and QDO/X-α-Gal/AbA media, the positive control (pGBKT7-53 + pGADT7-T) grew normally, whereas the negative control (pGBKT7-Lam + pGADT7-T) showed no growth, validating the reliability of the assay system.

Autoactivation assays revealed that on QDO and QDO/X-α-Gal/AbA media, only the BD constructs containing *S_2_-LbSLF5*, *S_2_-LbSLF8*, and *S_2_-LbSLF10* exhibited slight growth, indicating mild autoactivation activity for these specific BD plasmids. No autoactivation was detected for the remaining BD constructs or the corresponding empty vectors (Supplementary Fig. 1A). The AD construct harboring *LbSBP1* grew normally on DDO plates but failed to grow on QDO and QDO/X-α-Gal/AbA plates, demonstrating the absence of autoactivation for *LbSBP1* (Supplementary Fig. 1B). Following 4 days of incubation, the co-transformation of *LbSBP1* and *S_2_-LbSLF9* yielded only a few colonies on QDO plates, with no growth observed on QDO/X-α-Gal/AbA plates. After an additional 3 days of incubation, the colony number on the QDO plates increased with further expansion, whereas no growth was evident on the QDO/X-α-Gal/AbA plates (Supplementary Fig. 1C).
